# Reducing pain and anxiety with virtual reality in (outpatient) gynecological procedures: a systematic review with meta-analysis

**DOI:** 10.1016/j.xagr.2026.100640

**Published:** 2026-04-15

**Authors:** Annelotte van Haaps, Merel Oskam, Kim Dreyer, Kimmy Rosielle, Ralph de Vries, Ben Willem Mol, Madelon van Wely, Jan Willem Kallewaard, Anneke Schreurs, Velja Mijatovic

**Affiliations:** 1 Department of Reproductive Medicine, Amsterdam University Medical Centers, Vrije Universiteit Amsterdam, Amsterdam, The Netherlands (van Haaps, Dreyer, Rosielle, Schreurs, and Mijatovic); 2 Amsterdam Reproduction and Development research institute, Amsterdam University Medical Centers, Amsterdam, The Netherlands (van Haaps, Dreyer, Rosielle, Schreurs, and Mijatovic); 3 School of Medical Sciences, Vrije Universiteit Amsterdam, Amsterdam, The Netherlands (Oskam); 4 Medical Library, Vrije Universiteit Amsterdam, Amsterdam, The Netherlands (de Vries); 5 Department of Anesthesiology, Academic Medical Center, Amsterdam University Medical Centers, Amsterdam, The Netherlands (Kallewaard); 6 Department of Anesthesiology, Rijnstate Ziekenhuis, Arnhem, The Netherlands (Kallewaard); 7 Department of Reproductive Medicine, Academic Medical Center, Amsterdam University Medical Centers, Amsterdam, The Netherlands (van Wely); 8 Department of Obstetrics and Gynaecology, Monash University, Clayton, VIC, Australia (Mol); 9 Department of Gynaecology and Obstetrics, Aberdeen Centre for Women’s Health Research, School of Medicine, Medical Sciences and Nutrition, University of Aberdeen, Aberdeen, UK (Mol); 10 Department of Pediatrics, Onze Lieve Vrouwe Gasthuis, Amsterdam, The Netherlands (Oskam)

**Keywords:** anxiety, colposcopy, episiotomy repair, gynecological procedures, hysterosalpingography, hysteroscopy, intra-uterine device insertion, pain, systematic review, virtual reality

## Abstract

**Background:**

Virtual Reality (VR) is increasingly used in medicine for pain and anxiety management. It has been frequently studied in the field of Gynecology and Obstetrics and found effective during labour. However, its application during (outpatient) gynecological procedures as pain and anxiety management remains underexplored.

**Objective:**

This systematic review with meta-analysis aimed to study the effect of VR on pain, anxiety and patient experiences when provided during (outpatient) gynecological procedures.

**Study design:**

A systematic review was performed, by systematically searching PubMed, Embase, APA PsycInfo and Web of Science (Core Collection) from inception to March 20th 2024. Articles on the effect of VR on pain (VAS or NRS) and anxiety (all measurement tools) during (outpatient) gynecological procedures were included. Study designs had to be randomized controlled trials (RCTs) or cohort studies. Data were collected independently by two reviewers. A meta-analysis was performed to calculate mean differences (MD) with 95% Confidence Intervals (95%CI), Sensitivity analyses and a subgroup analysis were performed.

**Results:**

Thirteen RCTs, published between 2015 and 2024, reporting on VR during (outpatient) gynecological procedures for benign conditions were included. Virtual Reality significantly improved overall pain scores (MD -0.95 (95%CI -1.44 to -0.47); n=12, I^2^=77%; scale 0−10), preprocedural anxiety (MD -3.33 (95%CI -5.84 to -0.82); n=5, I^2^=5%; scale 0−100) and postprocedural anxiety (MD-15.83 (95%CI -29.39 to -2.17); n=6, I^2^=93%; scale0−100).

**Conclusion:**

The meta-analysis suggests that VR could positively influence overall pain and procedural anxiety. Not all studies reported on all outcome measures which might have introduced reporting bias and lead to an overestimation of the effect of VR. Future studies should further study the correlation between pain and anxiety and the effect of VR on this. However, VR provides an alternative to pain medication, without the risks associated with pharmaceuticals, making it an attractive option.

## Introduction

Originally developed for flight simulation, military training and entertainment, Virtual Reality (VR) was first used in the medical field in 1994 to simulate surgery and improve training for healthcare providers.^1^ Since then, VR has also been found effective in reducing patients’ pain and anxiety during medical procedures and has therefore been increasingly studied in the past decades.^2^^,^^3^ The core mechanism of VR lies in the ability to provide distraction by competing for the limited shared attentional resources with incoming nociceptive signals, thereby reducing procedural pain and anxiety.^4^ This is done by placing people in an artificial three-dimensional (3D) environment, often delivered by a head-mounted display (HMD) covering the eyes, which provides a combination of visual (and auditory) stimuli to ensure optimal VR immersiveness.^5^ A systematic review by Gupta et al.^6^ found that VR during medical procedures did not only provide distraction, but also enhanced the perception of control over pain, thereby increasing pain tolerance and threshold. However, it is important to acknowledge that pain perception is subjective and influenced by various factors, including psychological aspects such as anxiety.^7^ In the field of Gynecology and Obstetrics, JahaniShoorab et al.^8^ was the first who successfully applied VR as pain management during episiotomy repair. Thereafter, VR has been further studied and has mostly been found effective in reducing pain and/or anxiety during labour. However, evidence on the efficacy of VR during (outpatient) gynecological procedures for benign conditions is limited.^9^, ^10^, ^11^, ^12^

An association has been suggested between pain and anxiety. Craven et al.^13^ found an increased demand for pain medication among emergency department patients with severe anxiety, whereas Zhang et al.^14^ found preoperative anxiety a predictor of postoperative pain in patients undergoing laparoscopic hysterectomy. Moreover, a patients’ psychological state can impact sedation levels, where high anxiety can impede achieving optimal sedation. Finally, anxiety is associated with adverse psychological and somatic outcomes which can negatively affect patient satisfaction.^13^^,^^15^, ^16^, ^17^ This underlines the importance of broadening pain and anxiety management,^18^ where Virtual Reality could offer a non-pharmacological solution. However, evidence regarding VR during (outpatient) gynecological procedures is limited. Since these procedures are often associated with varying levels of anxiety and/or discomfort, VR might be beneficial. This systematic review aims to collect and study the use of VR during these procedures, evaluating its impact on anxiety, pain and patient experiences.

## Materials and method

This systematic review is reported in accordance with the Preferred Reporting Items for Systematic Reviews and Meta-Analysis (PRISMA) guidelines.^19^ The inclusion and exclusion criteria, type of studies, study selection, type of exposure, main outcomes and data were specified and prospectively registered in the PROSPERO international register of systematic reviews (CRD42022298250, registration date 4 January 2022).

### Search strategy

A systematic search was conducted to identify all relevant articles regarding the effect of VR on pain and anxiety management during (outpatient) gynecological procedures for benign conditions. We performed systematic searches in the bibliographic databases PubMed, Embase, APA PsycInfo and Web of Science (Core Collection), supported by a medical information specialist. All databases were last assessed on March 20th 2024. We additionally included a recently published study by our research team at the Amsterdam UMC, resulting in all studies from inception to June 2024 being collected. Although studies applying VR during pregnancy and/or labour were excluded, the term “Obstetrics” was added to the search to prevent an article from being missed. Article references were searched for additional relevant publications. Duplicate articles were removed. Appendix S1 shows the full search strategy.

### Eligibility criteria

Randomized controlled trials (RCTs), prospective and retrospective cohort studies who applied VR with a head-mounted device (HMD) during (outpatient) gynecological procedures and procedural pain and/or anxiety as their primary outcome were considered eligible. The field of Gynecology was defined as the medical field specialized in health of the female reproductive system which includes its development, diagnosis, prevention and therapy of associated diagnoses.^20^ Furthermore, we defined (outpatient) gynecological procedures for benign conditions as diagnostic or therapeutic interventions which were invasive but performed with minimal to no incision, needed minimal repair or entailed excision that did not significantly alter morphology or require repair. Device placement in natural cavities, subcutaneous implants or injections were also considered gynecological procedures.^21^ Included studies involved procedures with baseline pain scores between 4.0 and 5.5 (scale 0–10).^22^, ^23^, ^24^, ^25^, ^26^ Studies with other primary outcomes who made mention of pain and/or anxiety management during gynecological procedures with VR, were screened to avoid missing relevant data. We considered ultrasound a non- to minimally invasive diagnostic procedure and therefore excluded studies reporting on this. Abstract-only articles, studies not involving benign gynecological procedures and studies on VR in pregnancy, labor or an educational setting were excluded.

### Outcomes

The main outcome was pain experienced during an (outpatient) gynecological procedure. Pain scores had to be measured on the Visual Analogue Score (VAS, scale 0.0–10.0 cm or 0.0–100.0 mm) or Numeric Rating Scale (NRS, scale 0.0–10.0). Although the two measuring tools are not completely interchangeable, we included both since they are considered to have a strong level of agreement and there is a significant correlation between them.^27^ Procedural anxiety was chosen as secondary outcome, where all possible measurement tools that objectify anxiety were included. Other secondary outcomes focused on patient experiences including patient satisfaction, side effects and physical parameters (blood pressure, heart rate, oxygen saturation).

### Selection process and data assessment

Found articles were imported into the digital program Rayyan (www.rayyan.ai), creating a database.^28^ All articles were screened for inclusion by title and abstract by two independent reviewers. When an article was considered relevant, full text was obtained and screened. Only articles that fully met the inclusion criteria were included. When there was a difference in judgement, the two independent reviewers performed a consensus process where the article with its primary and secondary outcomes were discussed until consensus was reached. If necessary, a third and fourth independent researcher were consulted where the same process was performed. In case of missing data, we contacted the relevant research group, requesting completion. The Cochrane risk of bias tool was used to assess bias and quality of evidence.

### Statistical analysis

When at least two studies reported one of our predetermined outcomes, we averaged the results using random effects meta-analysis. The outcome measure reported by participants receiving VR (intervention) were compared to participants not receiving VR (control). Regarding pain, since several studies made the division between worst and overall pain scores we analyzed these separately. All pain scores were measured using NRS or VAS (scale 0–10 and 0–100). Their means and standard deviations (SD) were, where applicable, converted to a scale from 0.0 to 10.0. Medians with interquartile ranges (IQR) were transformed to a mean with SD. Anxiety scores were measured using the adjusted Amsterdam Preoperative Anxiety and Information Scale (APAIS, scale 3–15),^29^ 10-Point Likert Scale or Numeric Rating Scale (NRS, scale 0–10)^30^, ^31^, ^32^ and the State Anxiety Inventory (SAI, scale 20–80).^33^, ^34^, ^35^ All were converted to a scale from 0 to 100 by dividing the mean and SD of the anxiety scores by the highest number of their corresponding scale and multiply the result by 100. The same was done for patient satisfaction. In case of missing SD we used summary statistic level imputation by taking the average of the available observed variances.^36^

Meta-analysis was performed using Review Manager Version 5.4. All outcomes were analyzed using the unstandardized mean difference, to calculate a mean difference (MD) with 95% Confidence Interval (95%CI).^37^ By calculating I^2^, statistical heterogeneity was estimated. Sensitivity analyses were performed to assess the robustness of our findings to variations in key methodological and analytical decisions. First by calculating the standard mean difference (SMD) to correct for the different measuring tools among the studies. Second by excluding studies with high or unclear risk of bias and finally by pooling results using a fixed effects model. We performed a subgroup analysis where we differentiated between diagnostic, therapeutic and combined (diagnostic and therapeutic, e.g. biopsies) gynecological procedures. Finally, quality of our findings was assessed using the Grading of Recommendations Assessment, Development and Evaluation (GRADE) assessment.^38^

## Results

### Study characteristics

**Figure 1:**
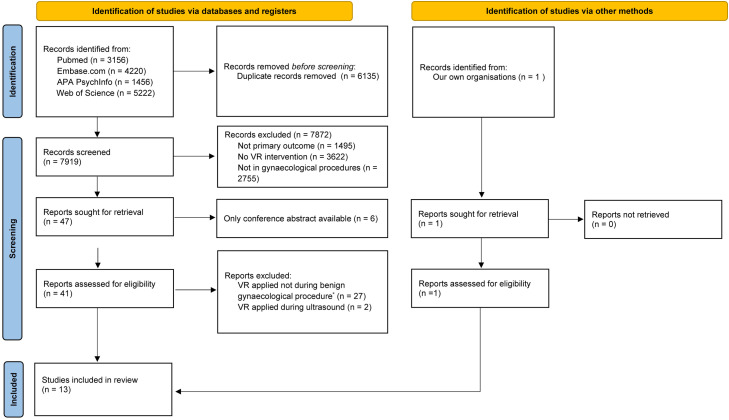
Flowchart of identification, screening and inclusion of articles on VR during gynecological procedures. *Excluded because VR was applied during caesarean section (n=3) or labor and delivery (n=12), during obstetric procedures like amniocentesis (n=7), to provide information or training (n=2), during procedures associated with gynecological cancer (n=2) or because it entailed a study protocol (n=1).

The literature search generated a total of 14.054 references (Figure 1). After removing duplicates, 7919 references remained. Screening led to a total of 13 studies being included.^8^^,^^26^^,^^29^, ^30^, ^31^, ^32^, ^33^, ^34^, ^35^^,^^39^, ^40^, ^41^, ^42^ All studies were RCTs and published between 2015 and June 2024. Virtual Reality was applied during office hysteroscopy (n=5), intra-uterine device (IUD) insertion (n=2), episiotomy repair postpartum (n=2), colposcopy (n=1) and hysterosalpingography (HSG) (n=3). A total of 564 patients underwent a gynecological procedure with VR and 543 patients without VR. All included studies are presented in Table 1.

**Table 1:** Included articles and their outcome measures.

Author, year of publication	Country of origin	Type of study	Number of participants	Type of gynaecological procedure	Standard treatment	Outcome measures in mean (SD)
JahaniShoorab et al. (2015)^8^	Iran	RCT	N = 30	Episiotomy repair	5ml lidocaine hydrochloride 2% solution	*Average pain score (NRP 0-100mm) during procedure:* during skin repair 16.7 (16.5) in VR group versus 39.3 (22.5) in control group. P-value main effect P<0.0001.
Deo et al. (2020)^30^	United Kingdom	RCT	N = 40	Hysteroscopy	Self-administration of analgesics prior to the procedure (paracetamol or NSAIDs)	*Average pain score (11-Point Likert scale 0-10) during procedure:* 3.70 (2.66) in VR group versus 6.00 (2.62) in control group (P=0.009). *Worst pain score (11-Point Likert scale 0-10) during procedure:* 5.65 (2.41) in VR group versus 7.85 (2.56) in control group (P=0.008). *Anxiety (11-Point Likert scale 0-10) during procedure:* 3.30 (2.03) in VR group versus 5.45 (3.35) in control group (P=0.019).
Brunn et al. (2021)^42^	United States of America	RCT	N = 50	Hysteroscopy	Self-administration of analgesics 1 hour prior to the procedure (800mg ibuprofen)	*Worst pain score (VAS, scale 0-100mm) during procedure:* 39.70 (28.40) in VR group versus 33.40 (28.40) in control group (P=0.317). *Average heart rate (Beats Per Minute, BPM) during procedure:* 79.2 (15.5) in VR group versus 79.2 (15.6) in control group (P=0.749). *Patient satisfaction (5-Point Likert Scale):* 18/25 very satisfied; 5/25 satisfied; 2/25 neutral in VR group versus 19/25 very satisfied; 5/25 satisfied; 1/25 neutral in control group.
Fouks et al. (2021)^41^	Israel	RCT	N = 82	Hysteroscopy	No analgesics	*Average pain score (NRS 0-10) during procedure:* 5.0 (3.0 – 8.0) in VR group versus 5.0 (3.0 – 7.2) in control group (P=0.670). *Average respiratory rate (RR) during procedure:* 14.0 (11.0 – 16.5) in VR group versus 14.0 (13.0 – 16.0) in control group (P=0.772). *Average pulse rate (PR) during procedure:* 80.0 (73.0 – 86.0) in VR group versus 81.5 (72.8 – 90.0) in control group (P=0.704).
Bal and Kulakaç (2023)^39^	Turkey	RCT	N = 122	Hysterosalpingography (HSG)	No analgesics	*Average pain score (VAS, scale 0-10cm) during procedure:* 8.24 (1.65) in VR group versus 8.50 (1.81) in control group (p=0.240).
Benazzouz et al. (2023)^32^	France	RCT	N = 100	Intra-uterine device (IUD) insertion	Pre-medication of choice (which included paracetamol, NSAIDs, phloroglucinol, tramadol or CBD)	*Average pain score (VAS, scale 0-10cm) during procedure:* 5.1 (2.5) in VR group versus 5.4 (2.7) in control group (p=0.54). Anxiety *(VAS, scale 0-10cm) before and during (reported immediately after) the procedure:* 4.8 (2.3) in VR group versus 4.7 (2.2) in control group before the procedure (p=0.82). Score of 4.2 (2.4) in VR group versus 4.8 (2.4) in control group during the procedure (p=0.13). *Patient satisfaction (VAS, scale 0-10cm):* 9.6 (1.1) in VR group versus 9.6 (1.1) in control group (p=0.87).
Hecken et al. (2023)^34^	Germany	RCT	N = 247	Colposcopy	No analgesics	*Average pain score (VAS, scale 0-100mm) during procedure:* 30 (10-50) in VR group versus 20 (10-50) in control group (P=0.65). *Anxiety (STAI-S questionnaire, scale 20-80) just before and after the procedure:* 42 (37-53.5) in VR group versus 46 (36-55) in control group before the procedure (p-value not reported). Score of 32 (16.5 – 49) in VR group versus 38 (23 – 52) in control group immediately after procedure (P=0.09). *Patient satisfaction (VAS, scale 0-100mm):* 100 (95-100) in VR group versus 100 (90-100) in control group (p=0.92). *Difference in average heart rate (Beats Per Minute, BPM) between baseline and end of colposcopy: -0.5 (8.3) in VR group versus -2.5 (6.5) in control group (P=0.21).*
Orhan and Bülez (2023)^26^	Turkey	RCT	N = 50	Episiotomy repair	5ml lidocaine hydrochloride 2% solution	*Average pain score (VAS, scale 0-10cm) during procedure:* during skin repair 2.24 (2.13) in VR group versus 4.36 (1.50) in control group (p=0.001). *Patient satisfaction (VAS, scale 0-10cm):* 9.72 (0.61) in VR group versus 7.06 (1.53) in control group (p=0.001).
Oz and Demirci (2023)^35^	Turkey	RCT	N = 80	Intra-uterine device (IUD) insertion	Clinics standard protocol	*Average pain score (VAS, scale 0-10cm) immediately after the procedure:* 0.47 (0.55) in VR group versus 2.12 (1.27) in control group (p=0.000). *Anxiety (STAI-S questionnaire, scale 20-80) just before and after the procedure:* 68.63 before the procedure versus 23.43 after the procedure in the VR group (p<0.05). Score of 66.78 before the procedure versus 61.25 after the procedure in the control group (p<0.05). *Patient satisfaction (5-Point Likert Scale):* VR application was appropriate to reduce pain and stress: 4.85 (0.36) in VR group.
Pelazas-Hernández et al. (2023)^40^	Spain	RCT	N = 154	Hysteroscopy	No analgesics	*Average pain score (VAS, scale 0-10cm) during procedure:* 2.45 (1.17) in VR group versus 3.97 (0.52) in control group (p=0.006). *Average heart rate (Beats Per Minute, BPM) during procedure):* 76.79 (11.14) in VR group versus 72.89 (13.49) in control group (p=0.237). *Blood pressure (mmHg) during procedure:* systolic pressure 133.13 (20.76) in VR group versus 137.46 (17.09) in control group (p=0.440). Diastolic pressure 76.86 (11.14) in VR group versus 77.23 (12.29) in control group (p=0.889). *Oxygen saturation (SpO_2_) during procedure:* 98.05 (1.75) in VR group versus 97.11 (2.25) in control group (p=0.732).
Sewell et al. (2023)^31^	United Kingdom	RCT	N = 83	Hysteroscopy	Self-administration of paracetamol and ibuprofen 1 hour prior to the procedure	*Average pain score (NRS, scale 0-10) during procedure:* 3.73 (2.39) in VR group versus 4.24 (3.07) in control group (p=0.41). *Worst pain score (NRS, scale 0-10) during procedure:* 5.32 (2.84) in VR group versus 5.07 (3.06) in control group (p=0.71). *Anxiety (NRS, scale 0-10) immediately after the procedure:* 3.29 (2.93) in VR group versus 4.79 (3.38) in control group (p=0.03). *Patient satisfaction (NRS, scale 0-10)*: 8.44 (2.27) in VR group versus 8.45 (2.27) in control group (p=0.50).
Sezer et al. (2023)^28^	Turkey	RCT	N = 62	Hysterosalpingography (HSG)	No analgesics	*Worst pain score (VAS, scale 0-10cm) during procedure:* 5.52 (2.40) in VR group versus 8.77 (1.84) in control group (P=0.000). *Anxiety (STAI questionnaire, scale 20-80) just before and after the procedure:* 39.42 (6.38) in VR group versus 41.93 (5.97) in control group (P=0.114) just before and 40.29 (6.49) in VR group versus 40.61 (6.29) in control group (P=0.843) immediately after the procedure. *Fear (VAS, scale 0-10cm) during procedure:* 3.71 (3.47) in VR group versus 8.26 (2.66) in control group (P=0.000). *Average pulse rate (PR) during procedure:* 90.94 (14.04) in VR group versus 84.39 (15.30) in control group (P= 0.084). *Blood pressure (mmHg) during procedure:* systolic pressure 119.06 (16.99) in VR group versus 122.32 (21.50) in control group (P=0.511). Diastolic pressure 80.71 (13.83) in VR group versus 81.06 (15.43) in control group (P=0.924). *Oxygen saturation (SpO_2_) during procedure:* 97.84 (1.34) in VR group versus 97.81 (0.94) in control group (P=0.913). *Patient satisfaction (VAS, scale 0-10cm)*: 8.16 (2.43) in VR group versus 6.62 (3.36) in control group (P=0.023).
Rosielle et al. (2023)^29^	The Netherlands	RCT	N = 134	Hysterosalpingography (HSG)	Self-administration of analgesics 24 and 2 hours prior to the procedure (NSAIDs)	*Average pain score (VAS, scale 0-10cm) during procedure:* 5.0 (2.10) in VR group versus 4.9 (2.13) in control group (P=0.915). *Worst pain score (VAS, scale 0-10cm) during procedure:* 6.8 (2.25) in VR group versus 6.6 (2.40) in control group (P=0.574). *Anxiety (in adjusted APAIS questionnaire, scale 3-15) before procedure:* 9.2 (2.3) in VR group versus 10.1 (2.4) in control group (P=0.018). *Mean patient satisfaction (5-Point Likert Scale):* 4.1 (0.84) in VR group versus 3.8 (1.02) in control group (P=0.197).

### VR technology

All studies applied VR during the entire gynecological procedure but several different VR systems and software were used across the included studies. In three studies, the head mounted device was connected to a video player, or a smartphone was placed in the VR glasses.^8^^,^^33^ In the other studies the VR intervention consisted of VR-glasses with a built-in display. Nine studies added relaxing auditory stimuli to the VR application. No studies included manual interactivity–remote controls adding hand movements into the VR experience (Table 2).

**Table 2:** The VR devices used in the included studies and applications installed on the VR devices.

Study	Type of VR device used	Type of VR content used	Duration of VR intervention
JahaniShoorab et al. (2015)^8^	A video player (3D Blu-ray/DVD player full HD, model BD660, Indonesia) connected to a pair of video glasses (Wrap 920 system, Vuzix factory, USA).	A 3D film (IMAX Dolphin and Whales 3D 1080p). Unknown whether sound was added to the VR experience.	Not described
Deo et al. (2020)^30^	VR headset “Oculus Go” with head-mounted display with built-in audio drivers.	An 8-minute video ‘Forest of Serenity’ commissioned by St Giles Hospice, developed by Holosphere and narrated by Sir David Attenborough. The immersive video simulated a calming rainforest and a lake setting with animated wildlife. Sound came from the VR headset.	The video was played for the duration of the outpatient hysteroscopy and replayed when the procedure exceeded 8 minutes. Patients were allowed to stop viewing the video or remove the headset at their own discretion or in the event of side effects.
Brunn et al. (2021)^42^	VR headset “Oculus Go” by Samsung with head-mounted display with built-in audio drivers.	The Guided Meditation VR App, with multiple environments and meditations. The VR experience was standardized by choosing the Nokia Spot 1 environment and the 10-minute guided Zen meditation. Sound came from the VR headset.	Not described.
Fouks et al. (2021)^41^	VR headset was a head-mounted display (SootheVR: AppliedVR, Los Angeles, California)	An immersive module of diving in a lagoon, with background sound effects, providing relaxing auditory stimuli.	During the entire hysteroscopy procedure.
Bal and Kulakaç (2023)^39^	VR glasses; exact type of headset not described.	Scenes from nature and a feeling of comfort and peace. Unknown whether sound was added to the VR experience.	During the entire HSG procedure
Benazzouz et al. (2023)^32^	VR headset was a head-mounted PICO (Qingdao Pico Technology Co, Ltd, China)	CE-certified software from Butterfly Therapeutics (Butterfly therapeutics ‘L’Effet Papillon’, Laval, France), application Bliss DTx. Four virtual universes of the forest, the meadow, the ocean floor or outer space. Use of an audio headset was optional.	During the entire procedure of IUD insertion.
Hecken et al. (2023)^34^	VR headset was a head-mounted PICO G2 4K (Qingdao Pico Technology Co, Ltd, China).	CE-certified software from SyncVR (SyncVR Medical DE GmbH, Murrhardt, Germany), application SyncVR Plug and Play – Virtual Reality Studio. A video of tropical forest and underwater swimming with dolphins. Unknown whether sound was added to the VR experience.	One group prior to the procedure (not included in the meta-analysis). One group both prior and during the procedure.
Orhan and Bülez (2023)^26^	Schulzz Vrg Pro 5-7 inch smartphone 3D VR glasses	Video images with relaxing music, determined by a search method from interactive platforms and based on expert opinion. Music came from the VR glasses.	During the entire episiotomy repair.
Oz and Demirci (2023)^35^	Everest VR0022 VR Box with an android mobile phone with headset placed inside the VR box.	Video of nature walk accompanied by music coming from the VR glasses.	During the entire procedure of IUD insertion.
Pelazas-Hernández et al. (2023)^40^	VR headset “Oculus Go” by Samsung with head-mounted display with built-in audio drivers.	VR application ‘A night sky’. No headphones were used, sounds came from the VR headset.	During the entire hysteroscopy procedure.
Sewell et al. (2023)^31^	VR headset was a head-mounted PICO G2.	CE-certified software from SyncVR (SyncVR Medical B.V., Utrecht, the Netherlands), application HypnoVR where ‘four seasons zen environment’ was played. Accompanying sound was played aloud without headphones.	During the entire hysteroscopy procedure.
Sezer et al. (2023)^28^	Virtual reality glasses (VR SHINECON), compatible with smartphones and playing 360-degree videos. A smartphone is placed in a closed chamber on the front of the device.	A video of the sea, underwater, and forest in addition to relaxing music.	During the entire HSG procedure.
Rosielle et al. (2023)^29^	VR headset was a head-mounted PICO G2 4K device (Pico Interactive Inc., San Francisco, United States of America)	CE-certified software from SyncVR (SyncVR Medical B.V., Utrecht, the Netherlands). Containing approx. twenty relaxing movies and breathing exercises, all designed for use in medical practice. Sound was switched off.	During the entire HSG procedure.

### Quality of evidence

**Figure 2:**
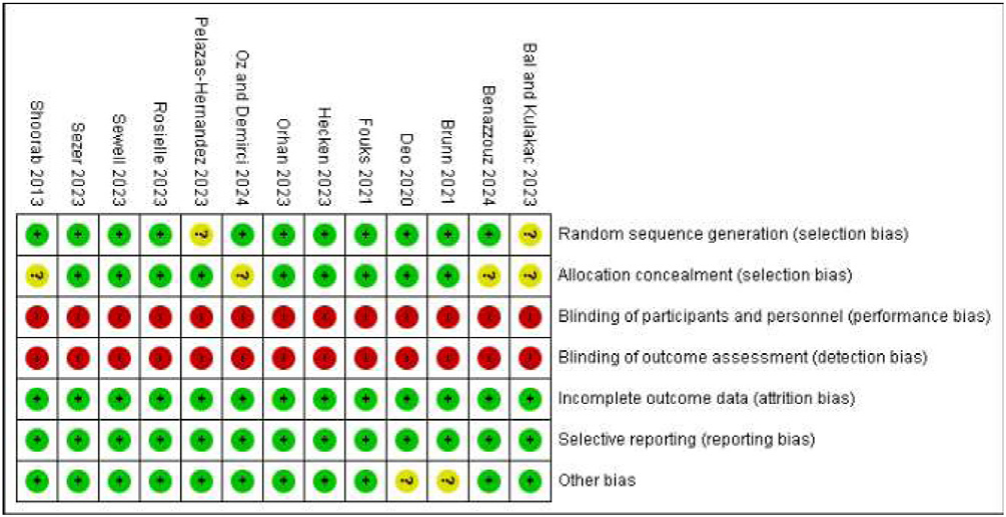
Risk of bias for all included studies. (A) Overall pain experienced during benign outpatient gynecological procedures. (B) Worst pain experienced during benign outpatient gynecological procedures

Eight studies were considered to have a low risk for selection bias.^8^^,^^26^^,^^29^, ^30^, ^31^^,^^33^^,^^34^^,^^41^ In five studies we considered selection bias to be unknown as they randomized from a list without allocation concealment, revealed the randomization outcome at least 48 hours prior to the procedure, or did not describe their allocation process nor provided this when requested.^8^^,^^32^^,^^35^^,^^39^^,^^40^ Since none of the studies were blinded, risk of performance- and detection bias was considered high.^8^^,^^26^^,^^29^, ^30^, ^31^, ^32^, ^33^, ^34^, ^35^^,^^39^, ^40^, ^41^, ^42^ Brunn et al.^42^ and Deo et al.^30^ mentioned the presence of recall bias but provided no further details. Therefore we assessed their “other bias” as unknown (Figure 2). When assessing the included studies on trustworthiness according to the Trustworthiness in RAndomised Controlled Trials (TRACT) checklist, no major concerns were found apart from the fact that some studies were retrospectively registered.^43^ No studies were therefore excluded (Appendix S2).

When assessing certainty of evidence according to the GRADE assessment, we considered it possible that the inability to blind participants and study personnel affected pain and/or anxiety. Due to high heterogeneity of data in all but preprocedural anxiety and the use of different measurement tools for pain and anxiety, we considered the level of inconsistency *serious* in overall pain, worst pain, pre and postprocedural anxiety. Since risk of other bias and imprecision were considered *not serious*, this led to certainty of evidence being moderate. In patient satisfaction and heart rate, certainty of data were considered high (Table 3).

**Table 3:** Evidence assessment on the different outcomes, according to the GRADE approach. Acquired using Review Manager 5.4 software.

Participants (studies) Follow-up	Risk of bias	Inconsistency	Indirectness	Imprecision	Overall certainty of evidence	Mean Difference (95%CI) with Virtual Reality
Average pain during outpatient benign gynaecological procedures						
1045 (12 RCTs)	not serious	serious^a^^,^^b^^,^^c^	not serious	not serious	⨁⨁⨁◯ Moderate^a^^,^^b^	MD **0.95 lower** (1.44 lower to 0.47 lower)
Worst pain during outpatient benign gynaecological procedures						
370 (5 RCTs)	not serious	serious^a^^,^^b^^,^^c^	not serious	not serious	⨁⨁⨁◯ Moderate^a^^,^^b^	MD **0.87 lower** (2.33 lower to 0.59 higher)
Pre-procedural anxiety during outpatient benign gynaecological procedures						
537 (5 RCTs)	not serious	serious^d^^,^^e^	not serious	not serious	⨁⨁⨁◯ Moderate^d^^,^^e^	MD **3.33 lower** (5.84 lower to 0.82 lower)
Post-procedural anxiety during outpatient benign gynaecological procedures						
525 (4 RCTs)	not serious	serious^c^^,^^d^^,^^e^	not serious	not serious	⨁⨁⨁◯ Moderate^c^^,^^d^^,^^e^	MD **15.83 lower** (29.49 lower to 2.17 lower)
Patient satisfaction during outpatient benign gynaecological procedures						
590 (6 RCTs)	not serious	not serious	not serious	not serious	⨁⨁⨁⨁ High	MD **0.82 higher** (0.2 lower to 1.84 higher)
Heart Rate during outpatient benign gynaecological procedures						
513 (5 RCTs)	not serious	not serious	not serious	not serious	⨁⨁⨁⨁ High	MD **2.1 higher** (0.31 higher to 3.9 higher)

a Some studies reported only on worst pain scores, where others reported only on average pain scores. Finally, some studies reported on both.

b Some studies found significant differences in pain scores, where others did not.

c High heterogeneity of data.

d Not all studies reported on anxiety scores.

e Some studies reported only on pre-procedural anxiety, where other reported only on post-procedural anxiety scores. Finally, some studies reported on both.

## Patient experiences during (outpatient) gynecological procedures

### Overall and worst pain

**Figure 3:**
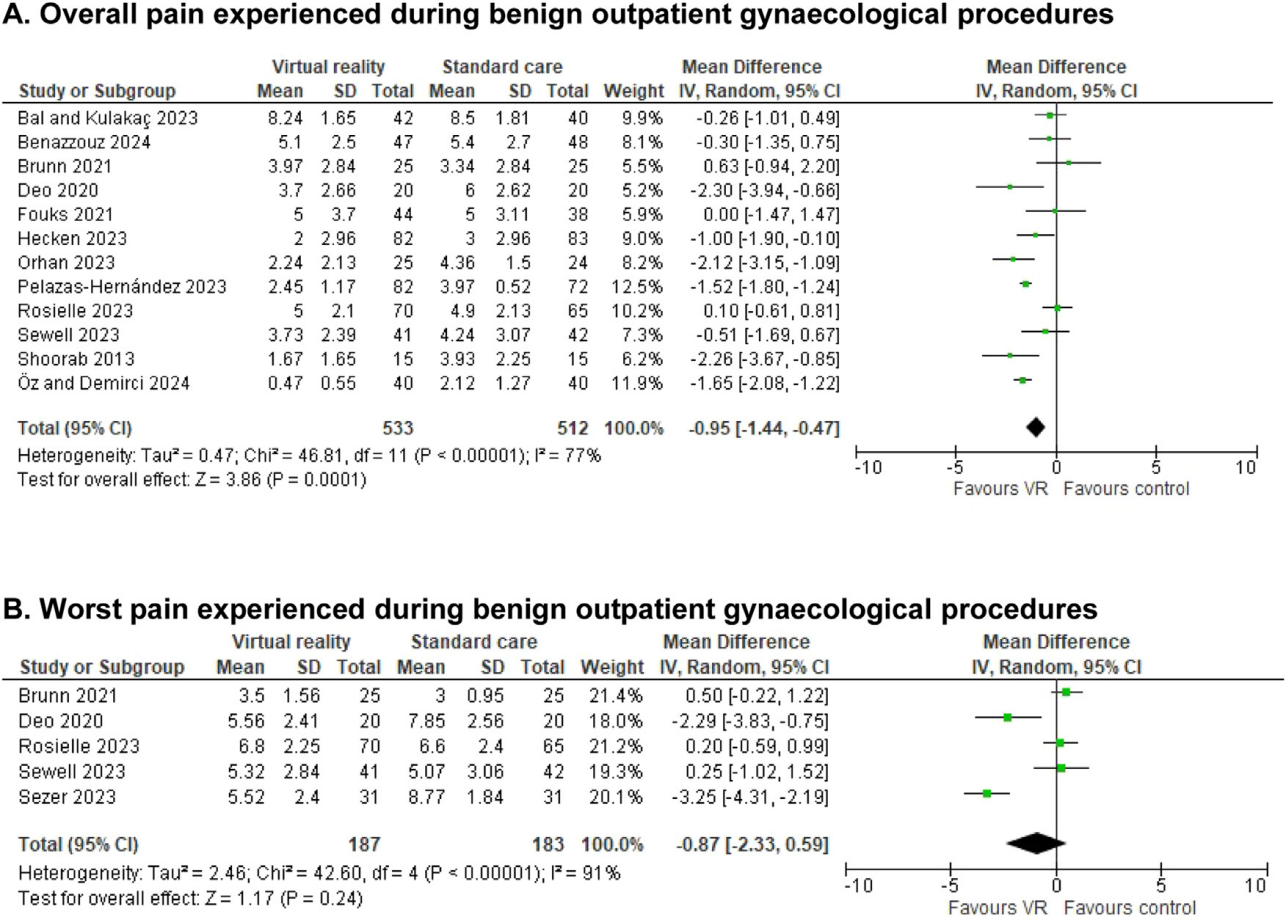
Reported overall and worst pain scores in the different studies of women undergoing either office hysteroscopy, intra-uterine device (IUD) insertion, episiotomy repair, colposcopy and hysterosalpingography (HSG). Scores were measured in VAS (scale 0–10 cm or 0–100 mm) or NRS (scale 0–10) and converted to a scale from 0 to 10. Forest plot of meta-analysis reporting on reported pain scores. (A) Overall pain score. (B) Worst pain score. Mean difference and 95% CI. MD<1 favor VR intervention, MD>1 favor control group. (A) Preprocedural anxiety measured during benign outpatient gynecological procedures. (B) Postprocedural anxiety measured during benign outpatient gynecological procedures.

All studies reported on procedural pain, either with overall pain scores,^8^^,^^26^^,^^32^^,^^34^^,^^35^^,^^39^, ^40^, ^41^ worst pain scores^33^ or both.^29^, ^30^, ^31^^,^^42^ A significant mean difference (MD) of −0.95 ((95%CI −1.44 to −0.47); n=12, I^2^=77%) in overall pain and a non-significant mean difference of −0.87 ((95%CI −2.33 to 0.59); n=5, I^2^=91%) in worst pain were seen in favor of VR. Both were measured on a scale from 0 to 10 (Figure 3). Expressed as standardized mean difference (SMD) these were −0.56 ((95% CI −0.94 to −0.18); I^2^=88%) for overall pain and −0.35 ((95% CI −0.98 to 0.28); I^2^=88%) for worst pain (Appendix Figure 1).

Pooling only data of studies with low risk of selection bias, the MD of overall pain scores was almost unchanged but lost significance (MD −0.74 (95%CI −1.52 to 0.04); n=8, I^2^=70%). When removing retrospectively registered studies, we saw no change in overall (MD −0.88 (95%CI −1.60 to −0.17); n=6, I^2^=79%) and worst pain (MD −0.93 (95% CI −3.19 to 1.33); n=3, I^2^=93%). Funnel plot analysis suggested a consistent negative effect across the studies but no publication bias or small-study effects (Appendix Figure 2). Due to high heterogeneity of data we performed sensitivity analyses, where worst pain scores became significant when using the fixed-effects model (Table 4).

**Table 4:** Sensitivity analysis using the fixed-effects model and the random-effects model. When negative numbers are displayed, the mean differences are in favour of VR.

Outcome measure	Random effects MD (95%CI)	Fixed effects MD (95%CI)
Average pain scores	-0.95 (-1.44, -0.47)	-1.25 (-1.44, -1.06)
Worst pain scores	-0.87 (-2.33, 0.59)	-0.43 (-0.86, -0.01)
Pre-procedural anxiety	-3.33 (-5.84, -0.82)	-3.36 (-5.77, -0.94)
Post-procedural anxiety	-15.83 (-29.49, -2.17)	-7.41 (-10.44, -4.37)
Patient satisfaction	0.82 (-0.20, 1.84)	0.73 (0.44, 1.02)
Heart rate	2.10 (0.31, 3.90)	2.10 (0.31, 3.89)

### Procedural anxiety

**Figure 4:**
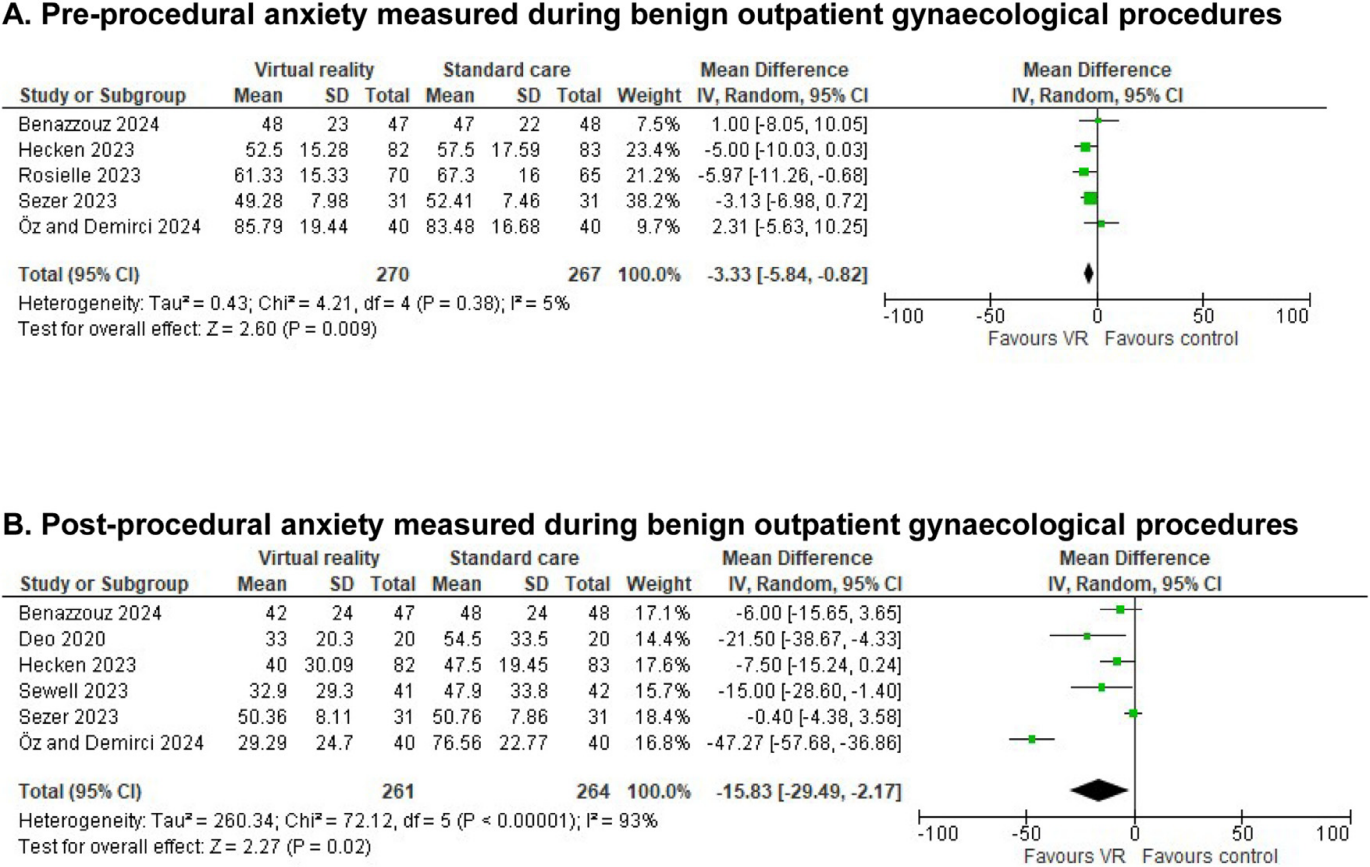
Reported anxiety scores in the different studies of women undergoing either office hysteroscopy, intra-uterine device (IUD) insertion, colposcopy and hysterosalpingography (HSG). Scores were measured in STAI, APAIS, NRS or 10-point Likert scale and converted to a scale from 0–100. Forest plot of meta-analysis reporting on reported anxiety scores. (A) Preprocedural anxiety. (B) Postprocedural anxiety. Mean difference and 95% CI. MD<1 favor VR intervention, MD>1 favor control group. Patient satisfaction Heart rate (HR; in BPM) measured during the procedures.

Seven studies evaluated procedural anxiety. Since it was measured either preprocedural,^29^ postprocedural^30^^,^^31^ or both,^32^, ^33^, ^34^, ^35^ these were analyzed separately (scale 0–100). Preprocedural anxiety (MD −3.33 (95%CI −5.84 to −0.82); n=4, I^2^=5%) and postprocedural anxiety (MD −15.83 (95%CI −29.49 to −2.17); n=5, I^2^=93%) showed a significant favorable effect of VR (Figure 4). Expressed as standardized mean difference (SMD) these were −0.20 ((95%CI −0.40 to 0.01); I^2^=29%) for preprocedural anxiety and −0.61 ((95%CI −1.10 to −0.12); I^2^=86%) for postprocedural anxiety (Appendix Figure 3).

After removing studies with an unknown risk of selection bias, the MD in preprocedural anxiety was unchanged (MD −4.36 (95%CI −7.00 to −1.71), n=3, I²=0%). In postprocedural anxiety, the MD decreased but remained significant (MD −8.50 (95%CI −16.97 to −0.04), n=4, I²=71%). When removing retrospectively registered studies, the MD became non-significant in postprocedural anxiety (MD-5.32 (95%CI −11.05 to 0.41); n=4, I^2^=52%). We performed a sensitivity analysis for postprocedural anxiety because of high heterogeneity of data, by analyzing data according to the fixed effects model (Table 4).

### Patient satisfaction

**Figure 5:**
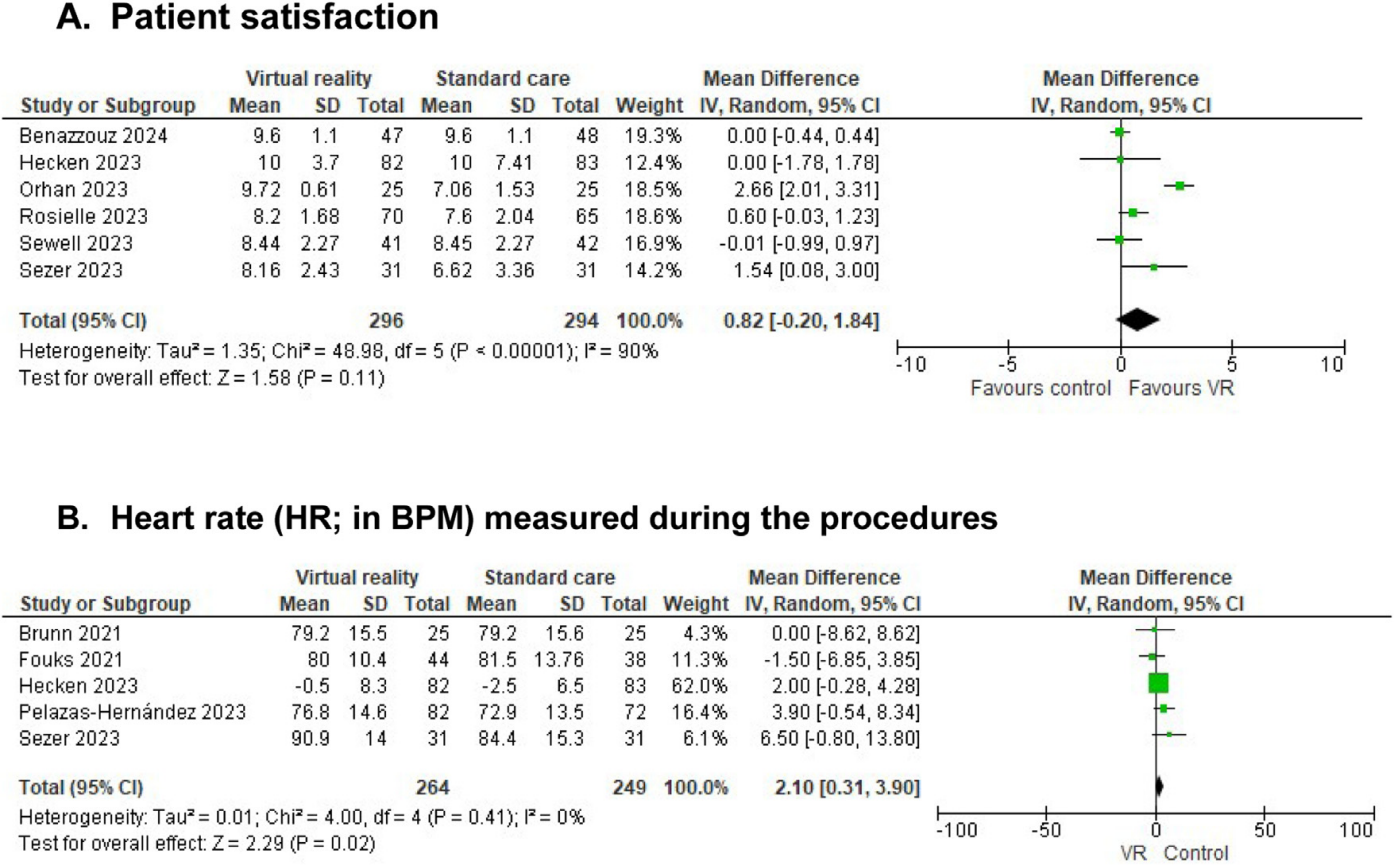
Reported patient satisfaction in NRS (scale 0–10) and heart rate measured during the procedures. Patient satisfaction was measured in the different studies of women undergoing either office hysteroscopy, intra-uterine device (IUD) insertion, episiotomy repair, colposcopy and hysterosalpingography (HSG). Heart rate (HR) was measured in studies with women undergoing either HSG or hysteroscopy measured in the different studies Forest plot of meta-analysis reporting on reported patient satisfaction. (A) Patient satisfaction. (B) Heart rate. Mean difference and 95% CI. MD<1 favor control group, MD>1 favor VR intervention.

In total, nine studies reported on patient satisfaction. Six studies reported on patient satisfaction using VAS, NRS or a 5-Point Likert Scale. No significant difference was seen in patient satisfaction, between intervention and control (MD 0.82 (95%CI −0.20 to 1.84); n=6, I^2^=90%. Scale 0–10) (Figure 5A). Three studies used interviews to objectify patient satisfaction (Table 5).

**Table 5:** Patient satisfaction reported during office hysteroscopy, during Intra-Uterine Device (IUD) insertion, during episiotomy repair, during colposcopy and during hysterosalpingography (HSG). If possible, P-values were reported.

Author, year of publication	Gynaecological procedure	Patient satisfaction	P-value
Deo et al. (2020)^30^	Hysteroscopy	**Semi-structured interviews. Positive experiences:** - Relaxation that distracted from pain -VR blocked sight of doctors and equipment - Pain not completely removed but distraction element aided in pain reduction and immediate recovery after instance of sharp pain - VR headset was comfortable **Negative experiences:** - No effect of VR technology - VR only effected during low to moderate pain - VR headset was not comfortable and claustrophobic (1/40 participants) - VR caused non-severe nausea (1/40 participants)	Not applicable
Brunn et al. (2021)^42^	Hysteroscopy	**Very satisfied**: 18/25 in VR group vs. 19/25 in control group. **Satisfied**: 5/25 in VR group vs. 5/25 in control group. **Neutral**: 2/25 in VR group vs. 1/25 in control group. **Unsatisfied**: 0/25 in VR group vs. 0/25 in control group. **Very unsatisfied**: 0/25 in VR group vs. 0/25 in control group. 88% found music very or slightly calming. 84% found medication very or slightly calming. 72% found VR environment very or slightly realistic.	Overall p-value: 1.000

Benazzouz et al. (2023)^32^	Intra-uterine device (IUD) insertion	Patient satisfaction (VAS, scale 0-10cm): 9.6 (1.1) in VR group versus 9.6 (1.1) in control group.	P=0.87
Hecken et al. (2023)^34^	Colposcopy	Patient satisfaction (VAS, scale 0-100mm): 100 (95-100) in VR group versus 100 (90-100) in control group.	P=0.92
Orhan and Bülez (2023)^26^	Episiotomy repair	Patient satisfaction (VAS, scale 0-10cm): 9.72 (0.61) in VR group versus 7.06 (1.53) in control group.	P=0.001
Öz and Demirci (2023)^35^	Intra-uterine device (IUD) insertion	- Watching a video with VR was effective and helpful in reducing stress (5-Point Likert scale): 4.83 (0.39) in VR group. - Watching a video with VR helped to focus attention elsewhere (5-Point Likert scale): 4.88 (0.34) in VR group. - I liked and was satisfied with the method of watching videos with the VR application: 4.88 (0.34) in VR group. - The video and music I watched with VR was motivating and helped me feel less pain: 4.83 (0.39) in VR group. - VR application was appropriate to reduce pain and stress: 4.85 (0.36) in VR group.	Not applicable.
Sewell et al. (2023)^31^	Hysteroscopy	Patient satisfaction (NRS, scale 0-10): 8.44 (2.27) in VR group versus 8.45 (2.27) in control group.	P=0.50
Sezer et al. (2023)^28^	Hysterosalpingo-graphy (HSG)	- Patient satisfaction (VAS 0-10cm) 15 minutes after HSG: 8.16 (SD 2.43) in VR group vs. 6.62 (3.36) in control group. - 87.1% of women wanted to use VR technology again if they had to undergo another HSG. 96.8% would recommend VR technology to other women undergoing an HSG.	P = 0.023 Not applicable
Rosielle et al. (2024)^29^	Hysterosalpingo-graphy (HSG)	- Patient satisfaction (5-Point Likert scale): 4.12 (0.86) in VR group vs. 3.78 (0.90) in control group. - Another HSG with VR (5-Point Likert scale): 3.43 (1.35) in VR group vs. 3.61 (1.37) in control group. - Another HSG without VR (5-Point Likert scale): 2.73 (1.25) in VR group vs. 3.63 (1.38) in control group. - Recommend VR technology to other women undergoing an HSG (5-Point Likert scale): 4.23 (0.91) in VR group. Not measured in the control group.	0.197 0.608 0.005 Not applicable

### Side effects

Side effects included nausea and/or vomiting, blurred vision and dizziness.^29^, ^30^, ^31^^,^^40^, ^41^, ^42^ They were short-lasting without serious consequences (Table 6). A meta-analysis could not be performed but the two studies comparing intervention to control found no statistical difference in side effects.^29^^,^^41^

**Table 6:** included studies and their reported side effects.

Author, year of publication	Side effects Virtual Reality	Side effects control group	P-value
JahaniShoorab et al. (2015)^8^	Not described	Not described	Not applicable
Deo et al. (2020)^30^	1/20 nausea during the procedure 1/20 pre-existing claustrophobia resulting in removal of VR intervention	No side effects No side effects	Not applicable Not applicable
Brunn et al. (2021)^42^	1/25 blurred vision during procedure, transient after removal of VR glasses	No side effects	Not applicable
Fouks et al. (2021)^41^	7/44 nausea or vomiting during the procedure 10/44 dizziness during the procedure	6/38 nausea or vomiting during the procedure 9/38 dizziness during the procedure	P=0.866 P=0.685
Bal and Kulakaç (2023)^39^	Not described	Not described	Not applicable
Benazzouz et al. (2023)^32^	Not described	Not described	Not applicable
Hecken et al. (2023)^34^	Not described	Not described	Not applicable
Orhan and Bülez (2023)^26^	Not described	Not described	Not applicable
Oz and Demirci (2023)^35^	Not described	Not described	Not applicable
Pelazas-Hernández et al. (2023)^40^	1/82 dizziness during the procedure	3/72 dizziness during the procedure	Not applicable
Sewell et al. (2023)^31^	2/41 mild nausea not requiring anti-emetics	1/42 mild nausea not requiring anti-emetics 1/42 pre-syncope 1/42 brief loss of consciousness from presumed vasovagal episode	
Sezer et al. (2023)^28^	Not described	Not described	Not applicable
Rosielle et al. (2023)^29^	Nausea average score of 1.6 (SD 1.08) * Dizziness average scores of 1.6 (SD 0.98) * Headache average score of 1.1 (SD 0.57) * Blurred vision average score of 1.3 (SD 0.84) *	Nausea average score of 1.7 (SD 1.18) * Dizziness average scores of 1.7 (SD 1.08) * Headache average score of 1.2 (SD 0.48) * Blurred vision average score of 1.2 (SD 0.41) *	P=0.422 P=0.583 P=0.482 P=0.489

⁎ Measured using the 5-Point Likert scale ranging from “1” representing none of the time to “5” representing all the time.

### Physical parameters

A meta-analysis could only be performed on the physical parameter of heart rate. A significant higher heart rate was seen in the control group (MD 2.10 (95%CI 0.31 to 3.90); n=5, I^2^=0%) (Figure 5B).

### Subgroup analyses

Only a subgroup analysis could be performed where studies reporting on the use of VR during diagnostic procedures^29^^,^^33^^,^^39^ were analyzed separately from studies during therapeutic procedures^8^^,^^26^^,^^32^^,^^35^ and studies during procedures where diagnostics were combined with small procedures like biopsies.^30^^,^^31^^,^^34^^,^^40^, ^41^, ^42^ Significant differences favoring VR were seen in overall pain for therapeutic and combined gynecological procedures, in preprocedural anxiety for diagnostic gynecological procedures and in postprocedural anxiety for combined gynecological procedures (Appendix Figure 4).

## Discussion

To the best of our knowledge, this is the first systematic review with meta-analysis evaluating the effect of VR on pain and anxiety during (outpatient) gynecological procedures for benign conditions. Our results suggest that VR could be contributory in reducing overall pain and anxiety, with subgroup analyses suggesting VR might be an effective tool to reduce overall pain in therapeutic and combined gynecological procedures, preprocedural anxiety in diagnostic procedures and postprocedural anxiety in combined gynecological procedures. Analysis with the fixed-effects model confirmed large variability across the included studies, confirming that an analysis with random-effects model was most appropriate. Based on the GRADE assessment, certainty of evidence was considered moderate.

### Strengths and limitations

Our review has several strengths. We established a clear research question and protocol, which was prospectively registered in PROSPERO. The review adhered to PRISMA guidelines. Data extraction and validation were done independently and blinded using Rayyan. Any discrepancies during article screening were meticulously addressed and if necessary a third or fourth independent researcher was consulted to reach consensus. In case of missing data or study design details, the relevant research group was contacted with the request of completing these. Trustworthiness of the included studies was assessed using the TRACT checklist. Finally, a subgroup- and sensitivity analysis were performed to ensure proper statistical analysis. Despite these strengths, our review also has several limitations. Not all studies reported on all outcome measures which might have introduced reporting bias with an overestimation of the effect of VR on the outcome measures. Publication bias might have contributed to this overestimation because articles with positive effects on an intervention are more likely to be published. Although studies without an effect of VR on pain and/or anxiety were included, this bias should be taken into account when interpreting the results. Furthermore, a considerable level of heterogeneity was seen in reported pain and postprocedural anxiety with potential influential factors such as participant characteristics, blinding, study quality, different measurement tools and different gynecological procedures where VR was applied. JahaniShoorab et al.^8^ reported a significant discrepancy in episiotomy depth between the VR and non-VR group, potentially contributing to data heterogeneity. The fact that only five out of thirteen studies had no analgesics added to the VR intervention, might have further contributed to data heterogeneity. Also because Orhan and Bülez and JahaniShoorab et al.,^8^ who provided Virtual Reality during episiotomy repair, did not make mention of labour analgesics such as an epidural. All included studies were randomized controlled trials, which might be explained by the fact that VR in medical procedures is a relatively new development and has not been widely studied. It is still to be determined in which medical area and on what procedure-associated factor it is most effective. The inability to use blinding due to the nature of the intervention may have introduced performance and detection bias. The Hawthorne effect might have been influential, where the knowledge of receiving an intervention affects pain- and/or anxiety scores.^44^

### Interpretation

Our meta-analysis showed a small but significant improvement in overall pain scores. It is debatable whether this difference is clinically relevant as Cepeda et al.^45^ considered a decrease of at least 1.3 in NRS units (20%) as relevant minimal pain reduction in moderate acute procedural pain. Since our findings fall below this threshold, VR may not provide clinically relevant pain reduction. This could be explained by suboptimal VR immersiveness, which is highlighted by previous studies as key to effective pain management.^5^^,^^46^, ^47^, ^48^, ^49^, ^50^, ^51^, ^52^ Furthermore, in a clinical setting VR is generally considered most effective for mild pain, where the distraction mechanism is not overridden by pain signals. In our subgroup analysis however, VR seemed to achieve clinically relevant pain reduction during therapeutic procedures, which are associated with higher pain levels compared to diagnostic procedures. The included studies did not provide a clear explanation for this finding, except the fact that administration of pain medication varied in studies providing VR during diagnostic procedures whereas all studies providing VR during therapeutic procedures applied pain medication. Furthermore, greater uncertainty associated with diagnostic and combined procedures might have affected experienced pain. Patients may feel they have more control during therapeutic procedures, as these are aimed at treating their condition. Diagnostic procedures are often associated with more uncertainty regarding the results and a potential diagnosis which can contribute to a greater sense of helplessness and lack of control, leading to increased procedural anxiety and ultimately procedural pain.^53^ We found that VR significantly reduced procedural anxiety, with the subgroup analysis finding it most effective when there was a diagnostic element. By providing distraction using VR, attention can be shifted from the medical procedure to pleasant or interesting stimuli, thereby reducing negative emotions such as anxiety.^54^, ^55^, ^56^ Furthermore, optimizing anxiety management with VR might also improve pain management, thereby transcending the clinically relevant threshold of 1.3 units. This is supported by the findings by Craven et al.^13^ and Zhang et al.^14^ where significant associations between pain and anxiety were seen.

We found no significant difference in patient satisfaction. It could be that relief over completion of the procedures resulted in no discernible difference in satisfaction scores between intervention and control. However, to our knowledge there are no studies supporting this and two of the included studies contradict this since they found significant better satisfaction scores when VR was applied.^26^^,^^33^ Patient satisfaction is crucial as it can strongly influence therapy compliance, reduce healthcare utilization, minimize malpractice litigation and contribute to a better prognosis.^57^ Healthcare providers increasingly recognize the importance of evaluating treatment innovations from a holistic approach which also considers improvements in other measures such as quality of life, medication use or patient satisfaction rather than solely symptom reduction.^58^ This approach considers the complexity of pain and its impact and evaluates improvements across a range of health domains that patients consider relevant by establishing minimal clinical important differences (MCIDs). Creating composite scores, where at least two outcome measures are combined into one single measure, can aid in evaluating the broader impact of health interventions.^58^^,^^59^ We recommend future studies to evaluate interventions from a holistic approach, ensuring that treatment efficacy is studied from all aspects.

## Conclusion

This systematic review with meta-analysis found a significant reduction in overall pain and procedural anxiety in favor of VR. Subgroup analysis suggests that VR might be more effective for overall pain during therapeutic procedures, and for procedural anxiety during procedures with a diagnostic element. The results should be interpreted cautiously due to data heterogeneity and potential bias. Future studies should explore the strong correlation between anxiety and perceived pain and apply a more holistic approach where the effect on composite health domains is considered much more important.

## Data availability

All data of the included studies and the extracted data used in the meta-analysis can be publicly assessed. Data will be made available to the editors of the journal pre and/or post publication for review or query upon request. The study was conducted according to PRISMA guidelines.

## CRediT authorship contribution statement

**Annelotte van Haaps:** Writing – original draft, Software, Formal analysis, Data curation, Conceptualization. **Merel Oskam:** Writing – review & editing, Data curation. **Kim Dreyer:** Writing – review & editing, Supervision, Conceptualization. **Kimmy Rosielle:** Writing – review & editing. **Ralph de Vries:** Methodology, Data curation. **Ben Willem Mol:** Writing – review & editing. **Madelon van Wely:** Writing – review & editing, Formal analysis. **Jan Willem Kallewaard:** Writing – review & editing. **Anneke Schreurs:** Writing – review & editing. **Velja Mijatovic:** Writing – review & editing, Supervision, Conceptualization.

## Appendix Supplementary materials

Image, application 1

Image, application 2

Image, application 3

## Footnotes

Supplementary material associated with this article can be found, in the online version, at doi:10.1016/j.xagr.2026.100640.
