# Supplementary material for: Reducing pain and anxiety with virtual reality in (outpatient) gynecological procedures: a systematic review with meta-analysis
Source: AJOG Glob Rep. 2026 Apr 15;6(2):100640. doi: 10.1016/j.xagr.2026.100640 (PMC13202560; doi:10.1016/j.xagr.2026.100640)
Supplement: Supplementary file 1 [file mmc1.docx]

**A. Overall pain experienced during benign outpatient gynaecological procedures**

**
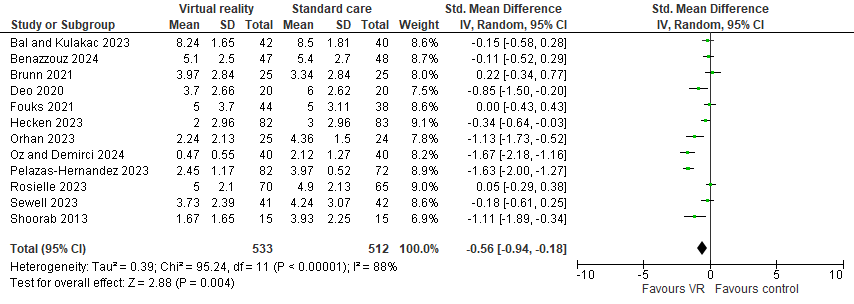
**

**B. Worst pain experienced during benign outpatient gynaecological procedures**

**
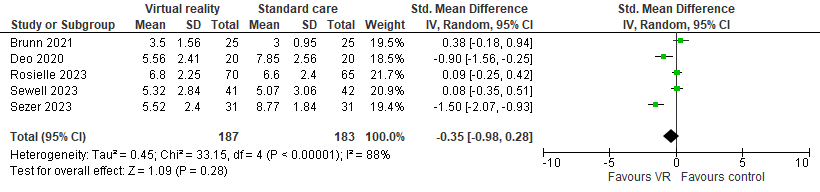
**

**Appendix Figure 1.** Reported overall and worst pain scores in the different studies of women undergoing either office hysteroscopy, Intra-Uterine Device (IUD) insertion, episiotomy repair, colposcopy and hysterosalpingography (HSG). Scores were measured in VAS (scale 0-10cm or 0-100mm) or NRS (scale 0-10) and converted to a scale from 0-10. Corrected for different measurement tools by measuring the standard mean difference (SMD) and 95% CI. MD<1 favour VR intervention, MD>1 favour control group.


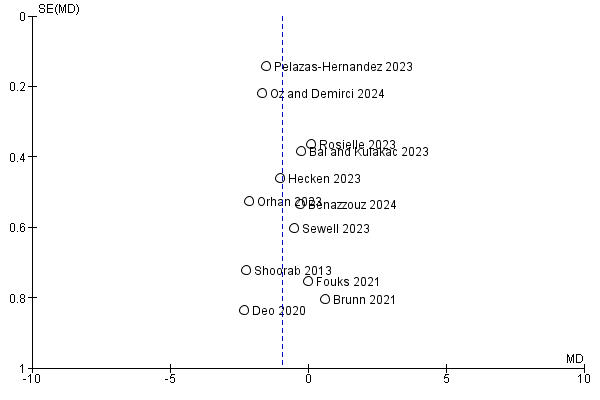


**Appendix Figure 2**. Funnel plot of comparison: Virtual reality versus standard treatment on average pain scores experienced during the procedure.

**A. Pre-procedural anxiety measured during benign outpatient gynaecological procedures**


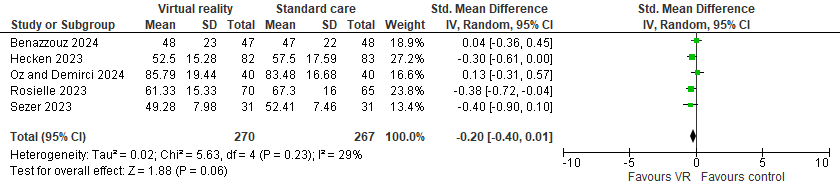


**B. Post-procedural anxiety measured during benign outpatient gynaecological procedures**

**
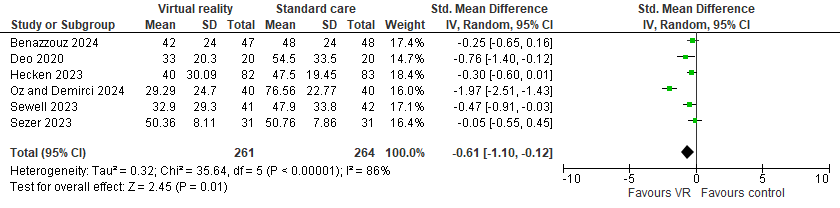
**

**Appendix Figure 3.** Reported anxiety scores in the different studies of women undergoing either office hysteroscopy, Intra-Uterine Device (IUD) insertion, colposcopy and hysterosalpingography (HSG). Scores were measured in STAI, APAIS, NRS or 10-point Likert scale and converted to a scale from 0-100. Forest plot of meta-analysis reporting on reported anxiety scores. (A) Pre-procedural anxiety. (B) Post-procedural anxiety. Corrected for different measurement tools by measuring the standard mean difference (SMD) and 95% CI. MD<1 favour VR intervention, MD>1 favour control group

**A. Overall pain experienced during interventional gynaecological procedures**


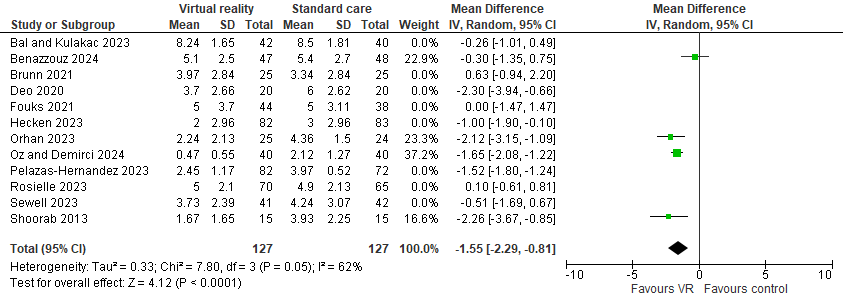


**B.** **Overall pain experienced during diagnostic gynaecological procedures**

**
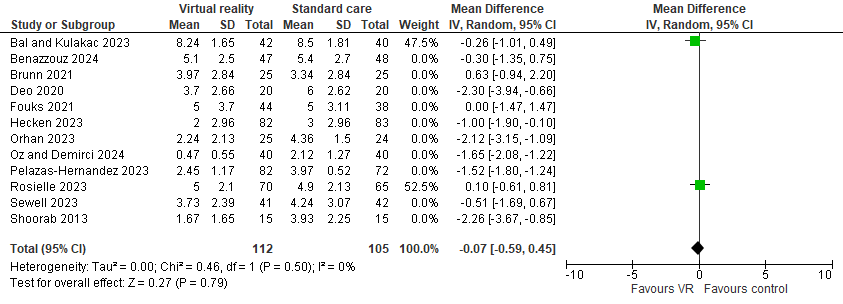
**

**C. Overall pain experienced during combined gynaecological procedures**

**
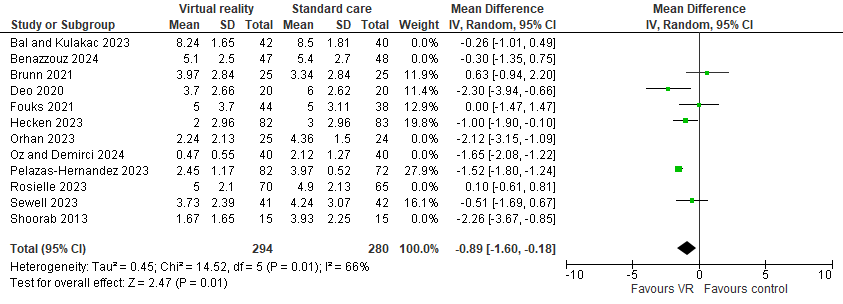
**

**D.** **Worst pain experienced during diagnostic gynaecological procedures**

**
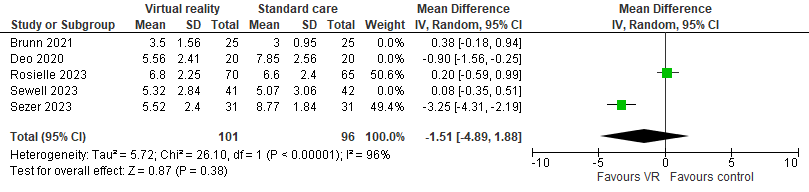
**

**E. Worst pain experienced during combined gynaecological procedures**

**
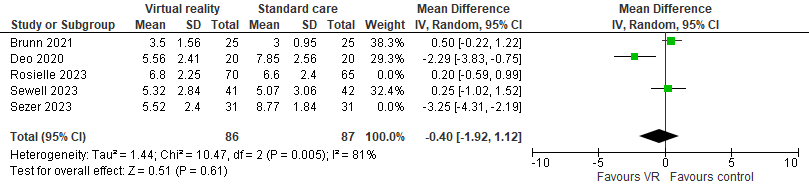
**

**F. Pre-procedural anxiety experienced during interventional gynaecological procedures**

**
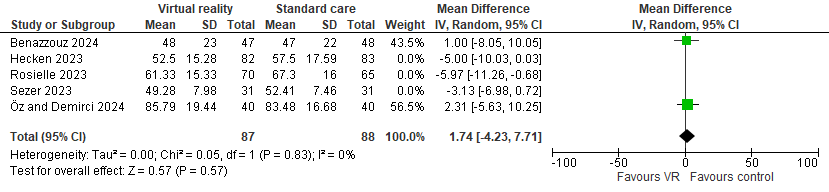
**

**G.** **Pre-procedural anxiety experienced during diagnostic gynaecological procedures**

**
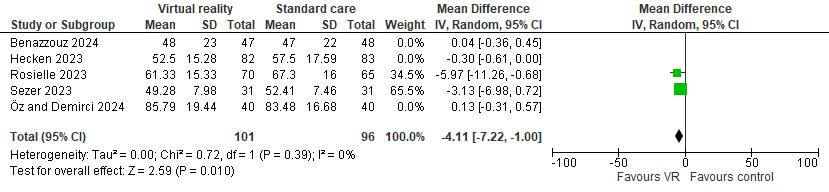
**

**G. Post-procedural anxiety experienced during interventional gynaecological procedures**


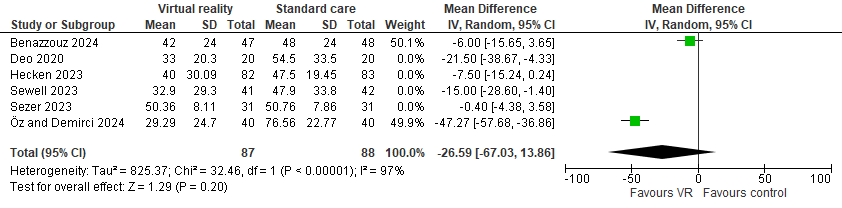


**H. Post-procedural anxiety experienced during combined gynaecological procedures**


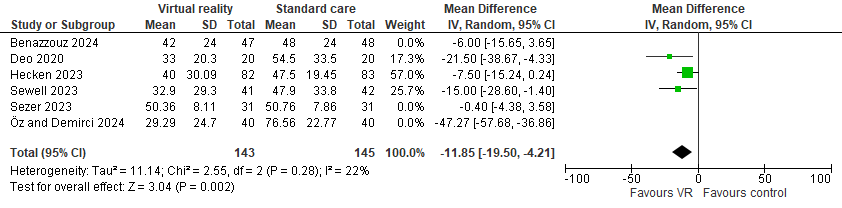


**Appendix Figure 4.** Subgroup analyses of reported pain scores and anxiety scores in the studies where women underwent either diagnostic procedures consisting of hysterosalpingography (HSG), interventional procedures consisting of Intra-Uterine Device (IUD) insertion and episiotomy repair, or a combined procedure consisting of office hysteroscopy or colposcopy. Forest plot of meta-analysis reporting on reported pain and anxiety scores. Mean difference and 95% CI. MD<1 favour VR intervention, MD>1 favour control group.
