# Supplementary material for: Reducing pain and anxiety with virtual reality in (outpatient) gynecological procedures: a systematic review with meta-analysis
Source: AJOG Glob Rep. 2026 Apr 15;6(2):100640. doi: 10.1016/j.xagr.2026.100640 (PMC13202560; doi:10.1016/j.xagr.2026.100640)
Supplement: Supplementary file 2 [file mmc2.docx]

**Appendix S1.** Search strategy.

**PubMed Session Results (20 Mar 2024)**

| Search | Query | Items found |
| --- | --- | --- |
| #6 | **#4 OR #5** | 3,156 |
| #5 | **#1 AND #3** | 2,512 |
| #4 | **#1 AND #2** | 720 |
| #3 | **"Patient Comfort"[Mesh] OR "Patient Satisfaction"[Mesh] OR "Pain Perception"[Mesh] OR "Pain Management"[Mesh] OR "Analgesia"[Mesh:NoExp] OR "Analgesia, Obstetrical"[Mesh] OR analgesia[tiab] OR ((stress[tiab] OR distress[tiab] OR pain[tiab] OR ache*[tiab] OR anxiety[tiab] OR anxious*[tiab] OR fear[tiab]) AND (management[tiab] OR reduc*[tiab] OR prevent*[tiab] OR relief[tiab] OR reliev*[tiab] OR alleviat*[tiab] OR perception*[tiab]))** | 1,032,558 |
| #2 | **"Gynecology"[Mesh] OR "Obstetrics"[Mesh] OR "Gynecologic Surgical Procedures"[Mesh] OR "Gynecological Examination"[Mesh] OR "Diagnostic Techniques, Obstetrical and Gynecological"[Mesh] OR "Obstetric Surgical Procedures"[Mesh] OR gynaecolog*[tiab] OR gynecolog*[tiab] OR obstetric*[tiab] OR "Pregnancy"[Mesh] OR "Pregnant Women"[Mesh] OR "Pregnancy Complications"[Mesh] OR "Preconception Care"[Mesh] OR pregnan*[tiab] OR gravidit*[tiab] OR gestation*[tiab] OR placentat*[tiab] OR prepregnan*[tiab] OR conception*[tiab] OR preconception*[tiab] OR labor[tiab] OR labour[tiab] OR birth[tiab]** | 1,721,094 |
| #1 | **"Virtual Reality"[Mesh] OR "Virtual Reality Exposure Therapy"[Mesh] OR "Augmented Reality"[Mesh] OR "virtual realit*"[tiab] OR "augmented realit*"[tiab] OR "mixed realit*"[tiab] OR "VR"[tiab] OR "virtual environment*"[tiab]** | 33,525 |

**Embase.com Session Results (20 Mar 2024)**

| Search | Query | Items found |
| --- | --- | --- |
| #7 | **#6 NOT ('conference abstract'/it OR 'conference review'/it)** | 4,220 |
| #6 | **#4 OR #5** | 5,489 |
| #5 | **#1 AND #3** | 4,123 |
| #4 | **#1 AND #2** | 1,540 |
| #3 | **'patient comfort'/exp OR 'patient satisfaction'/exp OR 'nociception'/exp OR 'analgesia'/de OR 'obstetric analgesia'/exp OR analgesia:ab,ti,kw OR ((stress:ab,ti,kw OR distress:ab,ti,kw OR pain:ab,ti,kw OR ache*:ab,ti,kw OR anxiety:ab,ti,kw OR anxious*:ab,ti,kw OR fear:ab,ti,kw) AND (management:ab,ti,kw OR reduc*:ab,ti,kw OR prevent*:ab,ti,kw OR relief:ab,ti,kw OR reliev*:ab,ti,kw OR alleviat*:ab,ti,kw OR perception*:ab,ti,kw))** | 1,542,199 |
| #2 | **'gynecology'/exp OR 'obstetrics'/exp OR 'gynecologic surgery'/exp OR 'gynecological examination'/exp OR 'obstetric procedure'/exp OR gynaecolog*:ab,ti,kw OR gynecolog*:ab,ti,kw OR obstetric*:ab,ti,kw OR 'pregnancy'/exp OR 'pregnant woman'/exp OR 'pregnancy complication'/exp OR pregnan*:ab,ti,kw OR gravidit*:ab,ti,kw OR gestation*:ab,ti,kw OR placentat*:ab,ti,kw OR prepregnan*:ab,ti,kw OR conception*:ab,ti,kw OR preconception*:ab,ti,kw OR labor:ab,ti,kw OR labour:ab,ti,kw OR birth:ab,ti,kw** | 2,376,122 |
| #1 | **'virtual reality'/exp OR 'virtual reality system'/exp OR 'virtual reality exposure therapy'/exp OR 'augmented reality'/exp OR 'augmented reality system'/exp OR 'virtual realit*':ab,ti,kw OR 'augmented realit*':ab,ti,kw OR 'mixed realit*':ab,ti,kw OR 'VR':ab,ti,kw OR 'virtual environment*':ab,ti,kw** | 53,182 |

**APA PsycInfo (Ebsco) Session Results (20 Mar 2024)**

| Search | Query | Items found |
| --- | --- | --- |
| S6 | **S4 OR S5** | 1,456 |
| S5 | **S1 AND S3** | 1,221 |
| S4 | **S1 AND S2** | 252 |
| S3 | **DE "Physical Comfort" OR DE "Client Satisfaction" OR DE "Pain Perception" OR DE "Pain Management" OR DE "Analgesia" OR TI (analgesia OR ((stress OR distress OR pain OR ache* OR anxiety OR anxious* OR fear) AND (management OR reduc* OR prevent* OR relief OR reliev* OR alleviat* OR perception*))) OR AB (analgesia OR ((stress OR distress OR pain OR ache* OR anxiety OR anxious* OR fear) AND (management OR reduc* OR prevent* OR relief OR reliev* OR alleviat* OR perception*))) OR KW (analgesia OR ((stress OR distress OR pain OR ache* OR anxiety OR anxious* OR fear) AND (management OR reduc* OR prevent* OR relief OR reliev* OR alleviat* OR perception*)))** | 239,183 |
| S2 | **DE "Gynecology" OR DE "Obstetrics" OR DE "Pregnancy" OR DE "Prenatal Care" OR TI (gynaecolog* OR gynecolog* OR obstetric* OR pregnan* OR gravidit* OR gestation* OR placentat* OR prepregnan* OR conception* OR preconception* OR labor OR labour OR birth) OR AB (gynaecolog* OR gynecolog* OR obstetric* OR pregnan* OR gravidit* OR gestation* OR placentat* OR prepregnan* OR conception* OR preconception* OR labor OR labour OR birth) OR KW (gynaecolog* OR gynecolog* OR obstetric* OR pregnan* OR gravidit* OR gestation* OR placentat* OR prepregnan* OR conception* OR preconception* OR labor OR labour OR birth)** | 214,117 |
| S1 | **DE "Virtual Reality" OR DE "Virtual Reality Exposure Therapy" OR DE "Augmented Reality" OR TI ("virtual realit*" OR "augmented realit*" OR "mixed realit*" OR "VR" OR "virtual environment*") OR AB ("virtual realit*" OR "augmented realit*" OR "mixed realit*" OR "VR" OR "virtual environment*") OR KW ("virtual realit*" OR "augmented realit*" OR "mixed realit*" OR "VR" OR "virtual environment*")** | 18,828 |

**Web of Science (Core Collection) Session Results (20 Mar 2024)**

| Search | Query | Items found |
| --- | --- | --- |
| #6 | **#4 OR #5** | 5,222 |
| #5 | **#1 AND #3** | 3,990 |
| #4 | **#1 AND #2** | 1,321 |
| #3 | **TS=("Patient Comfort" OR "Physical Comfort" OR "Patient Satisfaction" OR "Client Satisfaction" OR "nociception" OR "analgesia" OR (("stress" OR "distress" OR "pain" OR "ache*" OR "anxiety" OR "anxious*" OR "fear") AND ("management" OR "reduc*" OR "prevent*" OR "relief" OR "reliev*" OR "alleviat*" OR "perception*")))** | 1,437,762 |
| #2 | **TS=("gynaecolog*" OR "gynecolog*" OR "obstetric*" OR "pregnan*" OR "gravidit*" OR "gestation*" OR "placentat*" OR "prepregnan*" OR "conception*" OR "preconception*" OR "labor" OR "labour" OR "birth")** | 1,607,040 |
| #1 | **TS=("virtual realit*" OR "augmented realit*" OR "mixed realit*" OR "VR" OR "virtual environment*")** | 80,094 |
