# Supplementary material for: Reducing pain and anxiety with virtual reality in (outpatient) gynecological procedures: a systematic review with meta-analysis
Source: AJOG Glob Rep. 2026 Apr 15;6(2):100640. doi: 10.1016/j.xagr.2026.100640 (PMC13202560; doi:10.1016/j.xagr.2026.100640)
Supplement: Supplementary file 3 [file mmc3.docx]

**Table S1: The TRACT Screening Checklist**

| Article Title, Year | The effect of virtual reality on pain in primiparity women during episiotomy repair: a randomize clinical trial. |
| --- | --- |
| Author(s) | JahaniShoorab N., Ebrahimzadeh Zagami S., Nahvi A., Reza Mazluom S., Golmakani N., Talebi M., Pabarja F. |

Checklist

| *DOMAIN* | *ITEM* | *RATING* | | | *SUPPORT FOR JUDGEMENT* |
| --- | --- | --- | --- | --- | --- |
|  |  | No Concerns | Some Concerns/  No Information | Major Concerns |  |
| Governance | Absent or retrospective registration of RCTs. This is relevant for RCTs commencing after 2010 | Prospective registration. |  |  |  |
|  | Discrepancy of >15% between the intended sample size in the trial registration compared to the actual sample size achieved in the RCT | Discrepancy of < 15% |  |  |  |
|  | Absent or vague description of research ethics or apparent concerns regarding ethics | Thorough description of ethical concerns and research ethics obtained. |  |  |  |
| Author Group | Number of authors $\leq$3 or low author to study size ratio | Author group of 7. |  |  |  |
|  | Other studies of authors have been retracted not on request of the authors | No identifiable retracted studies, as checked by retractiondatabase.org. |  |  |  |
|  | Large number of RCTs published in a short time frame by one author/in one institute | Normal. |  |  |  |
| Plausibility of Intervention Usage | Insufficient or implausible description of allocation concealment (e.g. two interventions but only one placebo) |  | Allocation concealment described is plausible and reasonable but requires more detail. |  |  |
|  | Unnecessary or illogical description of methodological standards (e.g. use of sealed envelopes in a placebo-controlled trial) | Methodology ideal for study design. |  |  |  |
| Timeframe | Fast recruitment of participants within the study time (especially single centre studies) | 30 participants over a period of 3 months in a large Iranian center. Not unusually fast. |  |  |  |
|  | Short or impossible time frame between ending recruitment/follow up and submission of the paper (take into account time to outcome e.g. live birth, pregnancy outcome etc.) | Recruitment ended in July 2012, publication in May 2015. |  |  |  |
| Drop-Out Rates | Zero participants lost to follow up or no reasons mentioned for loss of follow up | Rationale for patients lost to follow-up provided. |  |  |  |
|  | Ideal number of losses to follow up resulting in perfectly rounded number in each group (e.g. groups of 50 or 100) | Not applicable for this study. |  |  |  |
| Baseline Characteristics | No or few baseline (<5) characteristics presented |  |  |  | No baseline characteristics provided. |
|  | Implausible patient characteristics judging from common sense, the literature and local data (e.g. similar standard deviations for completely different characteristics with different means and distributions) | Normal characteristics of labour and episiotomy. |  |  |  |
|  | Perfect balance for multiple baseline characteristics or significant/large differences between baseline characteristics | Not applicable for this study. |  |  |  |
|  | Important prognostic factors are not reported as baseline characteristics | Not applicable for this study. |  |  |  |
| Outcomes | Effect size that is much larger than in other RCTs regarding the same topic | Not applicable for this study. |  |  |  |
|  | Conflicting information between outcomes (e.g. more ongoing pregnancies than clinical pregnancies) | Not applicable for this study. |  |  |  |
|  | Change in primary outcome from registration to publication | Not applicable for this study. |  |  |  |

| Article Title, Year | Virtual Reality for acute pain in outpatient hysteroscopy: a randomised controlled trial. |
| --- | --- |
| Author(s) | Deo N., Khan K.S., Mak J., Allotey J., Gonzalez Carreras F.J., Fusari G., Benn J. |

Checklist

| *DOMAIN* | *ITEM* | *RATING* | | | *SUPPORT FOR JUDGEMENT* |
| --- | --- | --- | --- | --- | --- |
|  |  | No Concerns | Some Concerns/  No Information | Major Concerns |  |
| Governance | Absent or retrospective registration of RCTs. This is relevant for RCTs commencing after 2010 |  |  | Retrospective registration. |  |
|  | Discrepancy of >15% between the intended sample size in the trial registration compared to the actual sample size achieved in the RCT | Discrepancy of < 15% |  |  |  |
|  | Absent or vague description of research ethics or apparent concerns regarding ethics | Thorough description of ethical concerns and research ethics obtained. |  |  |  |
| Author Group | Number of authors $\leq$3 or low author to study size ratio | Author group of 7. |  |  |  |
|  | Other studies of authors have been retracted not on request of the authors | No identifiable retracted studies, as checked by retractiondatabase.org. |  |  |  |
|  | Large number of RCTs published in a short time frame by one author/in one institute | Normal. |  |  |  |
| Plausibility of Intervention Usage | Insufficient or implausible description of allocation concealment (e.g. two interventions but only one placebo) | Description of allocation concealment is detailed enough to replicate. |  |  |  |
|  | Unnecessary or illogical description of methodological standards (e.g. use of sealed envelopes in a placebo-controlled trial) | Methodology ideal for study design. |  |  |  |
| Timeframe | Fast recruitment of participants within the study time (especially single centre studies) | 40 participants over a period of 3 months in a large center in London. Based on the weekly number of women attending who could be approached. |  |  |  |
|  | Short or impossible time frame between ending recruitment/follow up and submission of the paper (take into account time to outcome e.g. live birth, pregnancy outcome etc.) | Recruitment ended in October 2018, publication in July 2020. |  |  |  |
| Drop-Out Rates | Zero participants lost to follow up or no reasons mentioned for loss of follow up | Rationale for patients lost to follow-up provided. |  |  |  |
|  | Ideal number of losses to follow up resulting in perfectly rounded number in each group (e.g. groups of 50 or 100) |  | 53 women eligible, 13 exclusion leading to 20/20 in both groups. |  |  |
| Baseline Characteristics | No or few baseline (<5) characteristics presented | An adequate amount of baseline characteristics that are relevant to the study. |  |  |  |
|  | Implausible patient characteristics judging from common sense, the literature and local data (e.g. similar standard deviations for completely different characteristics with different means and distributions) | Normal patient characteristics. |  |  |  |
|  | Perfect balance for multiple baseline characteristics or significant/large differences between baseline characteristics | Not applicable for this study. |  |  |  |
|  | Important prognostic factors are not reported as baseline characteristics | Not applicable for this study. |  |  |  |
| Outcomes | Effect size that is much larger than in other RCTs regarding the same topic | Not applicable for this study. |  |  |  |
|  | Conflicting information between outcomes (e.g. more ongoing pregnancies than clinical pregnancies) | Not applicable for this study. |  |  |  |
|  | Change in primary outcome from registration to publication | Not applicable for this study. |  |  |  |

| Article Title, Year | Virtual-Reality Effects on Acute Pain During Office Hysteroscopy: A Randomized Controlled Trial |
| --- | --- |
| Author(s) | Brunn E., Cheney M., Hazen N., Morozov V., Robinson J.K. |

Checklist

| *DOMAIN* | *ITEM* | *RATING* | | | *SUPPORT FOR JUDGEMENT* |
| --- | --- | --- | --- | --- | --- |
|  |  | No Concerns | Some Concerns/  No Information | Major Concerns |  |
| Governance | Absent or retrospective registration of RCTs. This is relevant for RCTs commencing after 2010 |  |  | Retrospective registration |  |
|  | Discrepancy of >15% between the intended sample size in the trial registration compared to the actual sample size achieved in the RCT | Discrepancy of < 15% |  |  |  |
|  | Absent or vague description of research ethics or apparent concerns regarding ethics | Thorough description of ethical concerns and research ethics obtained. |  |  |  |
| Author Group | Number of authors $\leq$3 or low author to study size ratio | Author group of 5. |  |  |  |
|  | Other studies of authors have been retracted not on request of the authors | No identifiable retracted studies, as checked by retractiondatabase.org. |  |  |  |
|  | Large number of RCTs published in a short time frame by one author/in one institute | Normal. |  |  |  |
| Plausibility of Intervention Usage | Insufficient or implausible description of allocation concealment (e.g. two interventions but only one placebo) | Description of allocation concealment is detailed enough to replicate. |  |  |  |
|  | Unnecessary or illogical description of methodological standards (e.g. use of sealed envelopes in a placebo-controlled trial) | Methodology ideal for study design. |  |  |  |
| Timeframe | Fast recruitment of participants within the study time (especially single centre studies) | 50 participants over a period of 7 months in a American center. Not unusually fast. |  |  |  |
|  | Short or impossible time frame between ending recruitment/follow up and submission of the paper (take into account time to outcome e.g. live birth, pregnancy outcome etc.) | Recruitment ended in November 2020, publication in June 2022. |  |  |  |
| Drop-Out Rates | Zero participants lost to follow up or no reasons mentioned for loss of follow up | Rationale for patients lost to follow-up provided. |  |  |  |
|  | Ideal number of losses to follow up resulting in perfectly rounded number in each group (e.g. groups of 50 or 100) | Not applicable for this study. | . |  |  |
| Baseline Characteristics | No or few baseline (<5) characteristics presented | An adequate amount of baseline characteristics that are relevant to the study. |  |  |  |
|  | Implausible patient characteristics judging from common sense, the literature and local data (e.g. similar standard deviations for completely different characteristics with different means and distributions) | Normal patient characteristics. |  |  |  |
|  | Perfect balance for multiple baseline characteristics or significant/large differences between baseline characteristics | Not applicable for this study. |  |  |  |
|  | Important prognostic factors are not reported as baseline characteristics | Not applicable for this study. |  |  |  |
| Outcomes | Effect size that is much larger than in other RCTs regarding the same topic | Not applicable for this study. |  |  |  |
|  | Conflicting information between outcomes (e.g. more ongoing pregnancies than clinical pregnancies) | Not applicable for this study. |  |  |  |
|  | Change in primary outcome from registration to publication | Not applicable for this study. |  |  |  |

| Article Title, Year | A virtual reality system for pain and anxiety management during outpatient hysteroscopy – A randomized control trial. |
| --- | --- |
| Author(s) | Fouks Y., Kern G., Cohen A., Reicher L., Shapira Z., Many A., Yogev Y., Rattan G. |

Checklist

| *DOMAIN* | *ITEM* | *RATING* | | | *SUPPORT FOR JUDGEMENT* |
| --- | --- | --- | --- | --- | --- |
|  |  | No Concerns | Some Concerns/  No Information | Major Concerns |  |
| Governance | Absent or retrospective registration of RCTs. This is relevant for RCTs commencing after 2010 |  |  | Retrospective registration |  |
|  | Discrepancy of >15% between the intended sample size in the trial registration compared to the actual sample size achieved in the RCT | Discrepancy of < 15% |  |  |  |
|  | Absent or vague description of research ethics or apparent concerns regarding ethics | Thorough description of ethical concerns and research ethics obtained. |  |  |  |
| Author Group | Number of authors $\leq$3 or low author to study size ratio | Author group of 8. |  |  |  |
|  | Other studies of authors have been retracted not on request of the authors | No identifiable retracted studies, as checked by retractiondatabase.org. |  |  |  |
|  | Large number of RCTs published in a short time frame by one author/in one institute | Normal. |  |  |  |
| Plausibility of Intervention Usage | Insufficient or implausible description of allocation concealment (e.g. two interventions but only one placebo) | Description of allocation concealment is detailed enough to replicate. |  |  |  |
|  | Unnecessary or illogical description of methodological standards (e.g. use of sealed envelopes in a placebo-controlled trial) | Methodology ideal for study design. |  |  |  |
| Timeframe | Fast recruitment of participants within the study time (especially single centre studies) | 82 participants over a period of 5 months in a Israeli center. Not unusually fast. |  |  |  |
|  | Short or impossible time frame between ending recruitment/follow up and submission of the paper (take into account time to outcome e.g. live birth, pregnancy outcome etc.) | Recruitment ended in August 2020, publication in November 2021. |  |  |  |
| Drop-Out Rates | Zero participants lost to follow up or no reasons mentioned for loss of follow up | Rationale for patients lost to follow-up provided. |  |  |  |
|  | Ideal number of losses to follow up resulting in perfectly rounded number in each group (e.g. groups of 50 or 100) | Not applicable for this study. | . |  |  |
| Baseline Characteristics | No or few baseline (<5) characteristics presented | An adequate amount of baseline characteristics that are relevant to the study. |  |  |  |
|  | Implausible patient characteristics judging from common sense, the literature and local data (e.g. similar standard deviations for completely different characteristics with different means and distributions) | Normal patient characteristics. |  |  |  |
|  | Perfect balance for multiple baseline characteristics or significant/large differences between baseline characteristics | Not applicable for this study. |  |  |  |
|  | Important prognostic factors are not reported as baseline characteristics | Not applicable for this study. |  |  |  |
| Outcomes | Effect size that is much larger than in other RCTs regarding the same topic | Not applicable for this study. |  |  |  |
|  | Conflicting information between outcomes (e.g. more ongoing pregnancies than clinical pregnancies) | Not applicable for this study. |  |  |  |
|  | Change in primary outcome from registration to publication | Not applicable for this study. |  |  |  |

| Article Title, Year | Effect of comfort theory-based nursing care on pain and comfort in women undergoing hysterosalpingography: a randomized controlled trial. |
| --- | --- |
| Author(s) | Bal S., Kulakaç O. |

Checklist

| *DOMAIN* | *ITEM* | *RATING* | | | *SUPPORT FOR JUDGEMENT* |
| --- | --- | --- | --- | --- | --- |
|  |  | No Concerns | Some Concerns/  No Information | Major Concerns |  |
| Governance | Absent or retrospective registration of RCTs. This is relevant for RCTs commencing after 2010 |  |  | Retrospective registration. |  |
|  | Discrepancy of >15% between the intended sample size in the trial registration compared to the actual sample size achieved in the RCT | Discrepancy of < 15% |  |  |  |
|  | Absent or vague description of research ethics or apparent concerns regarding ethics | Thorough description of ethical concerns and research ethics obtained. |  |  |  |
| Author Group | Number of authors $\leq$3 or low author to study size ratio |  | Author group of 2. |  |  |
|  | Other studies of authors have been retracted not on request of the authors | No identifiable retracted studies, as checked by retractiondatabase.org. |  |  |  |
|  | Large number of RCTs published in a short time frame by one author/in one institute | Normal. |  |  |  |
| Plausibility of Intervention Usage | Insufficient or implausible description of allocation concealment (e.g. two interventions but only one placebo) | Description of allocation concealment is detailed enough to replicate. |  |  |  |
|  | Unnecessary or illogical description of methodological standards (e.g. use of sealed envelopes in a placebo-controlled trial) | Methodology ideal for study design. |  |  |  |
| Timeframe | Fast recruitment of participants within the study time (especially single centre studies) | 122 participants over a period of 8 months in a Turkish center. Not unusually fast. |  |  |  |
|  | Short or impossible time frame between ending recruitment/follow up and submission of the paper (take into account time to outcome e.g. live birth, pregnancy outcome etc.) | Recruitment ended in April 2021, publication in August 2023. |  |  |  |
| Drop-Out Rates | Zero participants lost to follow up or no reasons mentioned for loss of follow up |  | Patients lost to follow-up with insufficient rationale provided. |  |  |
|  | Ideal number of losses to follow up resulting in perfectly rounded number in each group (e.g. groups of 50 or 100) | Not applicable for this study. |  |  |  |
| Baseline Characteristics | No or few baseline (<5) characteristics presented | An adequate amount of baseline characteristics that are relevant to the study. |  |  |  |
|  | Implausible patient characteristics judging from common sense, the literature and local data (e.g. similar standard deviations for completely different characteristics with different means and distributions) | Normal patient characteristics. |  |  |  |
|  | Perfect balance for multiple baseline characteristics or significant/large differences between baseline characteristics | Not applicable for this study. |  |  |  |
|  | Important prognostic factors are not reported as baseline characteristics | Not applicable for this study. |  |  |  |
| Outcomes | Effect size that is much larger than in other RCTs regarding the same topic | Not applicable for this study. |  |  |  |
|  | Conflicting information between outcomes (e.g. more ongoing pregnancies than clinical pregnancies) | Not applicable for this study. |  |  |  |
|  | Change in primary outcome from registration to publication | Not applicable for this study. |  |  |  |

| Article Title, Year | Effects of virtual reality on pain during intrauterine device insertions: a randomized controlled trial. |
| --- | --- |
| Author(s) | Benazzouz I., Bouhnik C., Chapron A., Esvan M., Lavoué V., Brun T. |

Checklist

| *DOMAIN* | *ITEM* | *RATING* | | | *SUPPORT FOR JUDGEMENT* |
| --- | --- | --- | --- | --- | --- |
|  |  | No Concerns | Some Concerns/  No Information | Major Concerns |  |
| Governance | Absent or retrospective registration of RCTs. This is relevant for RCTs commencing after 2010 | Prospective registration |  |  |  |
|  | Discrepancy of >15% between the intended sample size in the trial registration compared to the actual sample size achieved in the RCT | Discrepancy of < 15% |  |  |  |
|  | Absent or vague description of research ethics or apparent concerns regarding ethics |  | Absent description. |  |  |
| Author Group | Number of authors $\leq$3 or low author to study size ratio | Author group of 5. |  |  |  |
|  | Other studies of authors have been retracted not on request of the authors | No identifiable retracted studies, as checked by retractiondatabase.org. |  |  |  |
|  | Large number of RCTs published in a short time frame by one author/in one institute | Normal. |  |  |  |
| Plausibility of Intervention Usage | Insufficient or implausible description of allocation concealment (e.g. two interventions but only one placebo) | Description of allocation concealment is detailed enough to replicate. |  |  |  |
|  | Unnecessary or illogical description of methodological standards (e.g. use of sealed envelopes in a placebo-controlled trial) | Methodology ideal for study design. |  |  |  |
| Timeframe | Fast recruitment of participants within the study time (especially single centre studies) | 100 participants over a period of 19 months in a French center. Not unusually fast. |  |  |  |
|  | Short or impossible time frame between ending recruitment/follow up and submission of the paper (take into account time to outcome e.g. live birth, pregnancy outcome etc.) | Recruitment ended in April 2022, publication in November 2023. |  |  |  |
| Drop-Out Rates | Zero participants lost to follow up or no reasons mentioned for loss of follow up | Rationale for patients lost to follow-up provided. |  |  |  |
|  | Ideal number of losses to follow up resulting in perfectly rounded number in each group (e.g. groups of 50 or 100) |  | 100 women eligible, 1 exclusion leading to 50/50 in both groups. |  |  |
| Baseline Characteristics | No or few baseline (<5) characteristics presented | An adequate amount of baseline characteristics that are relevant to the study. |  |  |  |
|  | Implausible patient characteristics judging from common sense, the literature and local data (e.g. similar standard deviations for completely different characteristics with different means and distributions) | Normal patient characteristics. |  |  |  |
|  | Perfect balance for multiple baseline characteristics or significant/large differences between baseline characteristics | Not applicable for this study. |  |  |  |
|  | Important prognostic factors are not reported as baseline characteristics | Not applicable for this study. |  |  |  |
| Outcomes | Effect size that is much larger than in other RCTs regarding the same topic | Not applicable for this study. |  |  |  |
|  | Conflicting information between outcomes (e.g. more ongoing pregnancies than clinical pregnancies) | Not applicable for this study. |  |  |  |
|  | Change in primary outcome from registration to publication | Not applicable for this study. |  |  |  |

| Article Title, Year | Virtual Reality for Anxiety Reduction in Women Undergoing Colposcopy: A Randomized Controlled Trial. |
| --- | --- |
| Author(s) | Hecken J., Halagiera P., Rehman S., Tempfer C.B., Rezniczek G.A. |

Checklist

| *DOMAIN* | *ITEM* | *RATING* | | | *SUPPORT FOR JUDGEMENT* |
| --- | --- | --- | --- | --- | --- |
|  |  | No Concerns | Some Concerns/  No Information | Major Concerns |  |
| Governance | Absent or retrospective registration of RCTs. This is relevant for RCTs commencing after 2010 | Prospective registration |  |  |  |
|  | Discrepancy of >15% between the intended sample size in the trial registration compared to the actual sample size achieved in the RCT | Discrepancy of < 15% |  |  |  |
|  | Absent or vague description of research ethics or apparent concerns regarding ethics | Thorough description of ethical concerns and research ethics obtained. |  |  |  |
| Author Group | Number of authors $\leq$3 or low author to study size ratio | Author group of 5. |  |  |  |
|  | Other studies of authors have been retracted not on request of the authors | No identifiable retracted studies, as checked by retractiondatabase.org. |  |  |  |
|  | Large number of RCTs published in a short time frame by one author/in one institute | Normal. |  |  |  |
| Plausibility of Intervention Usage | Insufficient or implausible description of allocation concealment (e.g. two interventions but only one placebo) | Description of allocation concealment is detailed enough to replicate. |  |  |  |
|  | Unnecessary or illogical description of methodological standards (e.g. use of sealed envelopes in a placebo-controlled trial) | Methodology ideal for study design. |  |  |  |
| Timeframe | Fast recruitment of participants within the study time (especially single centre studies) | 247 participants over a period of 14 months in a German center. Not unusually fast. |  |  |  |
|  | Short or impossible time frame between ending recruitment/follow up and submission of the paper (take into account time to outcome e.g. live birth, pregnancy outcome etc.) | Recruitment ended in July 2022, publication in July 2023. |  |  |  |
| Drop-Out Rates | Zero participants lost to follow up or no reasons mentioned for loss of follow up | Rationale for patients lost to follow-up provided. |  |  |  |
|  | Ideal number of losses to follow up resulting in perfectly rounded number in each group (e.g. groups of 50 or 100) | Not applicable for this study. | . |  |  |
| Baseline Characteristics | No or few baseline (<5) characteristics presented | An adequate amount of baseline characteristics that are relevant to the study. |  |  |  |
|  | Implausible patient characteristics judging from common sense, the literature and local data (e.g. similar standard deviations for completely different characteristics with different means and distributions) | Normal patient characteristics. |  |  |  |
|  | Perfect balance for multiple baseline characteristics or significant/large differences between baseline characteristics | Not applicable for this study. |  |  |  |
|  | Important prognostic factors are not reported as baseline characteristics | Not applicable for this study. |  |  |  |
| Outcomes | Effect size that is much larger than in other RCTs regarding the same topic | Not applicable for this study. |  |  |  |
|  | Conflicting information between outcomes (e.g. more ongoing pregnancies than clinical pregnancies) | Not applicable for this study. |  |  |  |
|  | Change in primary outcome from registration to publication | Not applicable for this study. |  |  |  |

| Article Title, Year | The effect of virtual reality glasses applied during the episiotomy on pain and satisfaction: a single blind randomized controlled study. |
| --- | --- |
| Author(s) | Orhan M., Bülez A. |

Checklist

| *DOMAIN* | *ITEM* | *RATING* | | | *SUPPORT FOR JUDGEMENT* |
| --- | --- | --- | --- | --- | --- |
|  |  | No Concerns | Some Concerns/  No Information | Major Concerns |  |
| Governance | Absent or retrospective registration of RCTs. This is relevant for RCTs commencing after 2010 |  |  | Retrospective registration |  |
|  | Discrepancy of >15% between the intended sample size in the trial registration compared to the actual sample size achieved in the RCT | Discrepancy of < 15% |  |  |  |
|  | Absent or vague description of research ethics or apparent concerns regarding ethics | Thorough description of ethical concerns and research ethics obtained. |  |  |  |
| Author Group | Number of authors $\leq$3 or low author to study size ratio |  | Author group of 2. |  |  |
|  | Other studies of authors have been retracted not on request of the authors | No identifiable retracted studies, as checked by retractiondatabase.org. |  |  |  |
|  | Large number of RCTs published in a short time frame by one author/in one institute | Normal. |  |  |  |
| Plausibility of Intervention Usage | Insufficient or implausible description of allocation concealment (e.g. two interventions but only one placebo) | Description of allocation concealment is detailed enough to replicate. |  |  |  |
|  | Unnecessary or illogical description of methodological standards (e.g. use of sealed envelopes in a placebo-controlled trial) | Methodology ideal for study design. |  |  |  |
| Timeframe | Fast recruitment of participants within the study time (especially single centre studies) | 50 participants over a period of 6 months in a Turkish center with 5 midwives and 1 gynaecologist. Not unusually fast. |  |  |  |
|  | Short or impossible time frame between ending recruitment/follow up and submission of the paper (take into account time to outcome e.g. live birth, pregnancy outcome etc.) | Recruitment ended in December 2021, publication in June 2023. |  |  |  |
| Drop-Out Rates | Zero participants lost to follow up or no reasons mentioned for loss of follow up | Rationale for patients lost to follow-up provided. |  |  |  |
|  | Ideal number of losses to follow up resulting in perfectly rounded number in each group (e.g. groups of 50 or 100) |  | 51 providing informed consent, 1 exclusion leading to 25/25 in both groups. |  |  |
| Baseline Characteristics | No or few baseline (<5) characteristics presented | An adequate amount of baseline characteristics that are relevant to the study. |  |  |  |
|  | Implausible patient characteristics judging from common sense, the literature and local data (e.g. similar standard deviations for completely different characteristics with different means and distributions) | Normal patient characteristics. |  |  |  |
|  | Perfect balance for multiple baseline characteristics or significant/large differences between baseline characteristics | Not applicable for this study. |  |  |  |
|  | Important prognostic factors are not reported as baseline characteristics | Not applicable for this study. |  |  |  |
| Outcomes | Effect size that is much larger than in other RCTs regarding the same topic | Not applicable for this study. |  |  |  |
|  | Conflicting information between outcomes (e.g. more ongoing pregnancies than clinical pregnancies) | Not applicable for this study. |  |  |  |
|  | Change in primary outcome from registration to publication | Not applicable for this study. |  |  |  |

| Article Title, Year | The effect of virtual reality glasses applied during intrauterine device insertion on pain, anxiety and satisfaction: randomized controlled study. |
| --- | --- |
| Author(s) | Öz T., Demirci N. |

Checklist

| *DOMAIN* | *ITEM* | *RATING* | | | *SUPPORT FOR JUDGEMENT* |
| --- | --- | --- | --- | --- | --- |
|  |  | No Concerns | Some Concerns/  No Information | Major Concerns |  |
| Governance | Absent or retrospective registration of RCTs. This is relevant for RCTs commencing after 2010 |  |  | Retrospective registration |  |
|  | Discrepancy of >15% between the intended sample size in the trial registration compared to the actual sample size achieved in the RCT | Discrepancy of < 15% |  |  |  |
|  | Absent or vague description of research ethics or apparent concerns regarding ethics | Thorough description of ethical concerns and research ethics obtained. |  |  |  |
| Author Group | Number of authors $\leq$3 or low author to study size ratio |  | Author group of 2. |  |  |
|  | Other studies of authors have been retracted not on request of the authors | No identifiable retracted studies, as checked by retractiondatabase.org. |  |  |  |
|  | Large number of RCTs published in a short time frame by one author/in one institute | Normal. |  |  |  |
| Plausibility of Intervention Usage | Insufficient or implausible description of allocation concealment (e.g. two interventions but only one placebo) | Description of allocation concealment is detailed enough to replicate. |  |  |  |
|  | Unnecessary or illogical description of methodological standards (e.g. use of sealed envelopes in a placebo-controlled trial) | Methodology ideal for study design. |  |  |  |
| Timeframe | Fast recruitment of participants within the study time (especially single centre studies) | 80 participants over a period of 4 months in a large center in Istanbul. Not unusually fast. |  |  |  |
|  | Short or impossible time frame between ending recruitment/follow up and submission of the paper (take into account time to outcome e.g. live birth, pregnancy outcome etc.) | Recruitment ended in January 2023, publication in March 2024. |  |  |  |
| Drop-Out Rates | Zero participants lost to follow up or no reasons mentioned for loss of follow up | No lost to follow-up, because VR was only provided during the procedure. |  |  |  |
|  | Ideal number of losses to follow up resulting in perfectly rounded number in each group (e.g. groups of 50 or 100) |  | 85 assessed for eligibility, 5 exclusion leading to 40/40 in both groups. |  |  |
| Baseline Characteristics | No or few baseline (<5) characteristics presented | An adequate amount of baseline characteristics that are relevant to the study. |  |  |  |
|  | Implausible patient characteristics judging from common sense, the literature and local data (e.g. similar standard deviations for completely different characteristics with different means and distributions) | Normal patient characteristics. |  |  |  |
|  | Perfect balance for multiple baseline characteristics or significant/large differences between baseline characteristics | Not applicable for this study. |  |  |  |
|  | Important prognostic factors are not reported as baseline characteristics | Not applicable for this study. |  |  |  |
| Outcomes | Effect size that is much larger than in other RCTs regarding the same topic | Not applicable for this study. |  |  |  |
|  | Conflicting information between outcomes (e.g. more ongoing pregnancies than clinical pregnancies) | Not applicable for this study. |  |  |  |
|  | Change in primary outcome from registration to publication | Not applicable for this study. |  |  |  |

| Article Title, Year | The effect of Virtual Reality on the Reduction of Pain in Women with an Indication for Outpatient Diagnostic Hysteroscopy: A randomized controlled trial. |
| --- | --- |
| Author(s) | Pelazas-Hernández J.A., Varillas-Delgado D., González-Casado T., Cristóbal-Quevedo I., Alonso-Bermejo A., Ronchas-Martínez M., Cristóbal-García I. |

Checklist

| *DOMAIN* | *ITEM* | *RATING* | | | *SUPPORT FOR JUDGEMENT* |
| --- | --- | --- | --- | --- | --- |
|  |  | No Concerns | Some Concerns/  No Information | Major Concerns |  |
| Governance | Absent or retrospective registration of RCTs. This is relevant for RCTs commencing after 2010 | Prospective registration. |  |  |  |
|  | Discrepancy of >15% between the intended sample size in the trial registration compared to the actual sample size achieved in the RCT | Discrepancy of <15%. |  |  |  |
|  | Absent or vague description of research ethics or apparent concerns regarding ethics | Thorough description of ethical concerns and research ethics obtained. |  |  |  |
| Author Group | Number of authors $\leq$3 or low author to study size ratio | Author group of 7. |  |  |  |
|  | Other studies of authors have been retracted not on request of the authors | No identifiable retracted studies, as checked by retractiondatabase.org. |  |  |  |
|  | Large number of RCTs published in a short time frame by one author/in one institute | Not applicable for this author or institute. |  |  |  |
| Plausibility of Intervention Usage | Insufficient or implausible description of allocation concealment (e.g. two interventions but only one placebo) | Description of allocation concealment is detailed enough to replicate. |  |  |  |
|  | Unnecessary or illogical description of methodological standards (e.g. use of sealed envelopes in a placebo-controlled trial) | Methodology ideal for study design. |  |  |  |
| Timeframe | Fast recruitment of participants within the study time (especially single centre studies) | 152 participants over a period of 8 months in a large center in Madrid. Not unusually fast. |  |  |  |
|  | Short or impossible time frame between ending recruitment/follow up and submission of the paper (take into account time to outcome e.g. live birth, pregnancy outcome etc.) | Recruitment ended in October 2019, publication in May 2023. |  |  |  |
| Drop-Out Rates | Zero participants lost to follow up or no reasons mentioned for loss of follow up | Rationale for patients lost to follow-up provided |  |  |  |
|  | Ideal number of losses to follow up resulting in perfectly rounded number in each group (e.g. groups of 50 or 100) | Not applicable for this study. |  |  |  |
| Baseline Characteristics | No or few baseline (<5) characteristics presented | An adequate amount of baseline characteristics that are relevant to the study. |  |  |  |
|  | Implausible patient characteristics judging from common sense, the literature and local data (e.g. similar standard deviations for completely different characteristics with different means and distributions) | Normal patient characteristics. |  |  |  |
|  | Perfect balance for multiple baseline characteristics or significant/large differences between baseline characteristics | Not applicable for this study. |  |  |  |
|  | Important prognostic factors are not reported as baseline characteristics | Not applicable for this study. |  |  |  |
| Outcomes | Effect size that is much larger than in other RCTs regarding the same topic | Not applicable for this study. |  |  |  |
|  | Conflicting information between outcomes (e.g. more ongoing pregnancies than clinical pregnancies) | Not applicable for this study. |  |  |  |
|  | Change in primary outcome from registration to publication | Not applicable for this study. |  |  |  |

| Article Title, Year | Does Virtual Reality technology reduce pain and anxiety during outpatient hysteroscopy? A randomized controlled trial. |
| --- | --- |
| Author(s) | Sewell T., Fung Y., Al-Kufaishi A., Clifford K., Quinn S. |

Checklist

| *DOMAIN* | *ITEM* | *RATING* | | | *SUPPORT FOR JUDGEMENT* |
| --- | --- | --- | --- | --- | --- |
|  |  | No Concerns | Some Concerns/  No Information | Major Concerns |  |
| Governance | Absent or retrospective registration of RCTs. This is relevant for RCTs commencing after 2010 | Prospective registration. |  |  |  |
|  | Discrepancy of >15% between the intended sample size in the trial registration compared to the actual sample size achieved in the RCT | Discrepancy of <15%. |  |  |  |
|  | Absent or vague description of research ethics or apparent concerns regarding ethics | Thorough description of ethical concerns and research ethics obtained. |  |  |  |
| Author Group | Number of authors $\leq$3 or low author to study size ratio | Author group of 5. |  |  |  |
|  | Other studies of authors have been retracted not on request of the authors | No identifiable retracted studies, as checked by retractiondatabase.org. |  |  |  |
|  | Large number of RCTs published in a short time frame by one author/in one institute | One article per year maximum. |  |  |  |
| Plausibility of Intervention Usage | Insufficient or implausible description of allocation concealment (e.g. two interventions but only one placebo) | Description of allocation concealment is detailed enough to replicate. |  |  |  |
|  | Unnecessary or illogical description of methodological standards (e.g. use of sealed envelopes in a placebo-controlled trial) | Methodology ideal for study design. |  |  |  |
| Timeframe | Fast recruitment of participants within the study time (especially single centre studies) | 83 participants over a period of 8 months in a large center in London. Not unusually fast. |  |  |  |
|  | Short or impossible time frame between ending recruitment/follow up and submission of the paper (take into account time to outcome e.g. live birth, pregnancy outcome etc.) | Recruitment ended in October 2022, publication in May 2023. |  |  |  |
| Drop-Out Rates | Zero participants lost to follow up or no reasons mentioned for loss of follow up | No lost to follow-up, because VR was only provided during the procedure. |  |  |  |
|  | Ideal number of losses to follow up resulting in perfectly rounded number in each group (e.g. groups of 50 or 100) | Not applicable for this study. |  |  |  |
| Baseline Characteristics | No or few baseline (<5) characteristics presented | An adequate amount of baseline characteristics that are relevant to the study. |  |  |  |
|  | Implausible patient characteristics judging from common sense, the literature and local data (e.g. similar standard deviations for completely different characteristics with different means and distributions) | Normal patient characteristics. |  |  |  |
|  | Perfect balance for multiple baseline characteristics or significant/large differences between baseline characteristics | Not applicable for this study. |  |  |  |
|  | Important prognostic factors are not reported as baseline characteristics | Not applicable for this study. |  |  |  |
| Outcomes | Effect size that is much larger than in other RCTs regarding the same topic | Not applicable for this study. |  |  |  |
|  | Conflicting information between outcomes (e.g. more ongoing pregnancies than clinical pregnancies) | Not applicable for this study. |  |  |  |
|  | Change in primary outcome from registration to publication | Not applicable for this study. |  |  |  |

| Article Title, Year | The effect of virtual reality on women's perceived pain, fear, anxiety, and views about the procedure during hysterosalpingography: A randomized controlled trial. |
| --- | --- |
| Author(s) | Yilmaz Sezer N., Nazli Aker M., Gönenç I.M., Topuz Ş., Emre Şükür Y. |

Checklist

| *DOMAIN* | *ITEM* | *RATING* | | | *SUPPORT FOR JUDGEMENT* |
| --- | --- | --- | --- | --- | --- |
|  |  | No Concerns | Some Concerns/  No Information | Major Concerns |  |
| Governance | Absent or retrospective registration of RCTs. This is relevant for RCTs commencing after 2010 | Prospectively registration. |  |  |  |
|  | Discrepancy of >15% between the intended sample size in the trial registration compared to the actual sample size achieved in the RCT | Discrepancy of < 15% |  |  |  |
|  | Absent or vague description of research ethics or apparent concerns regarding ethics | Thorough description of ethical concerns and research ethics obtained. |  |  |  |
| Author Group | Number of authors $\leq$3 or low author to study size ratio | Author group of 5. |  |  |  |
|  | Other studies of authors have been retracted not on request of the authors | No identifiable retracted studies, as checked by retractiondatabase.org. |  |  |  |
|  | Large number of RCTs published in a short time frame by one author/in one institute | Normal. |  |  |  |
| Plausibility of Intervention Usage | Insufficient or implausible description of allocation concealment (e.g. two interventions but only one placebo) | Description of allocation concealment is detailed enough to replicate. |  |  |  |
|  | Unnecessary or illogical description of methodological standards (e.g. use of sealed envelopes in a placebo-controlled trial) | Methodology ideal for study design. |  |  |  |
| Timeframe | Fast recruitment of participants within the study time (especially single centre studies) | 62 participants over a period of 3 months in a Turkish center. Not unusually fast. |  |  |  |
|  | Short or impossible time frame between ending recruitment/follow up and submission of the paper (take into account time to outcome e.g. live birth, pregnancy outcome etc.) | Recruitment ended in June 2022, publication in July 2023. |  |  |  |
| Drop-Out Rates | Zero participants lost to follow up or no reasons mentioned for loss of follow up | Rationale for patients lost to follow-up provided. |  |  |  |
|  | Ideal number of losses to follow up resulting in perfectly rounded number in each group (e.g. groups of 50 or 100) | Not applicable for this study. | . |  |  |
| Baseline Characteristics | No or few baseline (<5) characteristics presented | An adequate amount of baseline characteristics that are relevant to the study. |  |  |  |
|  | Implausible patient characteristics judging from common sense, the literature and local data (e.g. similar standard deviations for completely different characteristics with different means and distributions) | Normal patient characteristics. |  |  |  |
|  | Perfect balance for multiple baseline characteristics or significant/large differences between baseline characteristics | Not applicable for this study. |  |  |  |
|  | Important prognostic factors are not reported as baseline characteristics | Not applicable for this study. |  |  |  |
| Outcomes | Effect size that is much larger than in other RCTs regarding the same topic |  | Pain scores during HSG for control group are higher than found in European studies. |  |  |
|  | Conflicting information between outcomes (e.g. more ongoing pregnancies than clinical pregnancies) | Not applicable for this study. |  |  |  |
|  | Change in primary outcome from registration to publication | Not applicable for this study. |  |  |  |

| Article Title, Year | No pain relief by virtual reality during hysterosalpingography (HSG): results from a randomized controlled trial |
| --- | --- |
| Author(s) | Rosielle K., van Haaps A.P., Kuijper E.A.M., Tonch N., Karim D.E.N.B., Oskam M.A., van den IJssel R., Mol B.W.J., Lambalk C.B., Dreyer K., Mijatovic V. |

Checklist

| *DOMAIN* | *ITEM* | *RATING* | | | *SUPPORT FOR JUDGEMENT* |
| --- | --- | --- | --- | --- | --- |
|  |  | No Concerns | Some Concerns/  No Information | Major Concerns |  |
| Governance | Absent or retrospective registration of RCTs. This is relevant for RCTs commencing after 2010 | Prospective registration |  |  |  |
|  | Discrepancy of >15% between the intended sample size in the trial registration compared to the actual sample size achieved in the RCT | Discrepancy of < 15% |  |  |  |
|  | Absent or vague description of research ethics or apparent concerns regarding ethics | Thorough description of ethical concerns and research ethics obtained. |  |  |  |
| Author Group | Number of authors $\leq$3 or low author to study size ratio | Author group of 11. |  |  |  |
|  | Other studies of authors have been retracted not on request of the authors | No identifiable retracted studies, as checked by retractiondatabase.org. |  |  |  |
|  | Large number of RCTs published in a short time frame by one author/in one institute | Normal. |  |  |  |
| Plausibility of Intervention Usage | Insufficient or implausible description of allocation concealment (e.g. two interventions but only one placebo) | Description of allocation concealment is detailed enough to replicate. |  |  |  |
|  | Unnecessary or illogical description of methodological standards (e.g. use of sealed envelopes in a placebo-controlled trial) | Methodology ideal for study design. |  |  |  |
| Timeframe | Fast recruitment of participants within the study time (especially single centre studies) | 135 participants over a period of 21 months in a Dutch center. Not unusually fast. |  |  |  |
|  | Short or impossible time frame between ending recruitment/follow up and submission of the paper (take into account time to outcome e.g. live birth, pregnancy outcome etc.) | Recruitment ended in October 2022, publication in September 2024. |  |  |  |
| Drop-Out Rates | Zero participants lost to follow up or no reasons mentioned for loss of follow up | Rationale for patients lost to follow-up provided. |  |  |  |
|  | Ideal number of losses to follow up resulting in perfectly rounded number in each group (e.g. groups of 50 or 100) | Not applicable for this study. | . |  |  |
| Baseline Characteristics | No or few baseline (<5) characteristics presented | An adequate amount of baseline characteristics that are relevant to the study. |  |  |  |
|  | Implausible patient characteristics judging from common sense, the literature and local data (e.g. similar standard deviations for completely different characteristics with different means and distributions) | Normal patient characteristics. |  |  |  |
|  | Perfect balance for multiple baseline characteristics or significant/large differences between baseline characteristics | Not applicable for this study. |  |  |  |
|  | Important prognostic factors are not reported as baseline characteristics | Not applicable for this study. |  |  |  |
| Outcomes | Effect size that is much larger than in other RCTs regarding the same topic | Not applicable for this study. |  |  |  |
|  | Conflicting information between outcomes (e.g. more ongoing pregnancies than clinical pregnancies) | Not applicable for this study. |  |  |  |
|  | Change in primary outcome from registration to publication | Not applicable for this study. |  |  |  |
